# Equivalent-circuit-based multi-objective parameter design and Pareto analysis of an LCC-S wireless power transfer system

**DOI:** 10.1371/journal.pone.0355130

**Published:** 2026-10-06

**Authors:** Gang Zhou, Shishi Liao, Qi Jiang

**Affiliations:** 1 School of Computer Science, Sichuan University Jinjiang College, Meishan, China; 2 School of Electrical and Electronic Information Engineering, Sichuan University Jinjiang College, Meishan, China; 3 Intelligent Digital Media Technology Key Laboratory of Sichuan Province, Chengdu, China; Northeastern University, CHINA

## Abstract

Parameter selection in an LCC-S compensated wireless power transfer system is difficult because the circuit variables are coupled and an increase in output power does not necessarily lead to higher transmission efficiency. In this study, the primary compensation inductance, equivalent load resistance, and mutual inductance were treated as three design variables, and their selection was formulated as a two-objective optimization problem. The search was performed using an archive-based particle swarm optimization method that combines dynamic inertia adjustment, probabilistic velocity pausing, and stagnation reset. MOPSO, NSGA-II, and MOGWO were used as comparison methods in 10 independent runs. Hypervolume, Spacing, the number of nondominated solutions, and runtime were recorded. Relative to MOPSO, the proposed method increased mean hypervolume by 8.45%, reduced Spacing by 41.14%, and reduced the observed MATLAB runtime by 26.30%. The obtained Pareto front contained candidates corresponding to a high-output-power boundary, an intermediate power–efficiency trade-off, and high-efficiency operation. Three representative candidates were further examined using a switching-level Simulink model together with model-calculated current and loss indicators. These results are intended to support preliminary parameter selection for LCC-S systems; hardware calibration is still required before the reported candidates can be used as practical operating points.

## 1. Introduction

Wireless power transfer (WPT) enables contactless energy delivery through electromagnetic coupling and resonant energy conversion [1,2]. It has been investigated for electric-vehicle charging [3–5], implantable medical devices [6–8], and consumer, mobile, or aerial applications [9–11]. Recent studies have further examined dynamic wireless charging for electric vehicles [12] and intermittent charging methods for cardiac pacemakers [13]. WPT research also encompasses radiative energy-transfer technologies [14], while artificial-intelligence methods are increasingly being explored for WPT system analysis and design [15]. In resonant WPT systems, output power and transmission efficiency depend jointly on the compensation topology, magnetic coupling, load condition, and parasitic losses. For LCC-S compensated systems, these factors are strongly coupled, and independent adjustment of individual circuit parameters may shift the operating point away from the desired resonance condition or produce an unfavorable power–efficiency trade-off. Joint parameter selection can therefore be formulated as a constrained multi-objective design problem.

Several optimization methods have been used in WPT system design. Genetic algorithms have been applied to component selection in LCC-S systems [16]. Particle swarm optimization (PSO) [17] has also been used because of its relatively simple implementation and population-based search capability, although premature convergence remains a known limitation [18]. Multi-objective methods are useful when output power and transmission efficiency must be considered together [19,20]. For the problem studied here, the main difficulty is that the compensation parameter, load condition, and magnetic coupling condition affect both objectives simultaneously.

Existing PSO variants address different parts of this problem. Adaptive or dynamic PSO changes algorithm parameters during the iteration process [21]. VPPSO [22] reduces the frequency of velocity updates, whereas MOPSO [23] uses an external archive to retain nondominated solutions. The quality of a multi-objective solution set can be evaluated using indicators such as hypervolume (HV) [24], which considers both convergence and the extent of the obtained Pareto front. In this study, these established PSO mechanisms are integrated into an archive-based Pareto parameter-design framework tailored to the LCC-S WPT problem, and the resulting procedure is referred to as Velocity-Pausing and Stagnation-Reset Particle Swarm Optimization (VPSR-PSO). The methodological contribution is therefore the coordinated application and evaluation of these mechanisms for the coupled power–efficiency design problem rather than the introduction of fundamentally new general-purpose optimization operators. Table 1 summarizes the relationship between the present framework and the PSO methods from which its main search mechanisms are derived.

**Table 1:** Relationship between representative particle swarm optimization methods and the present design framework.

Method	Main feature	Relevance to the present work
Standard PSO [17]	Uses particle position and velocity updates for global search.	Provides the basic particle-position and velocity-update framework on which the present LCC-S multi-objective procedure is built.
Adaptive/dynamic PSO [21]	Adjusts inertia weight or algorithm parameters during iteration.	Combines dynamic inertia adjustment with a Pareto-archive-based multi-objective framework.
VPPSO [22]	Introduces velocity pausing to improve global optimization performance.	Extends the velocity-pausing idea to the efficiency–power Pareto optimization of LCC-S WPT systems.
MOPSO [23]	Extends PSO to multi-objective optimization using an external Pareto archive.	Provides the Pareto-archive-based multi-objective framework into which dynamic inertia adjustment, velocity pausing, and stagnation reset are integrated for the present LCC-S design problem.

The aim of this study was to develop and evaluate an equivalent-circuit-based multi-objective parameter-design procedure for an LCC-S compensated WPT system. The primary compensation inductance, equivalent load resistance, and mutual inductance were jointly optimized, with output power and transmission efficiency treated as competing objectives. An archive-based particle swarm procedure integrating dynamic inertia adjustment, velocity pausing, and stagnation reset was used as the search method. The procedure was evaluated over 10 independent runs against representative multi-objective optimizers, and selected Pareto-front candidates were further examined using time-domain switching simulations and model-level electrical-stress indicators. The results show that the proposed framework can identify candidate parameter sets corresponding to different power–efficiency design preferences under the tested model assumptions.

## 2. System modeling and transmission characteristic analysis

This section establishes the equivalent-circuit model and transmission-characteristic analysis of the LCC-S compensated WPT system, providing the computational basis for the subsequent multi-objective optimization.

### 2.1. System modeling

Among various compensation topologies, the Series-Series (SS) topology is simple but sensitive to load variations [25]. The double-sided LCC topology offers excellent misalignment tolerance but requires more passive components, increasing system size and cost [26]. In contrast, the LCC-S topology combines the benefits of constant current output and a compact secondary side, making it highly suitable for EV charging applications [27,28].

The two-coil Magnetically-Coupled Resonant Wireless Power Transfer (MCRWPT) system is illustrated in Fig 1. The AC mains are first rectified through an AC–DC converter, converting the electrical energy into a DC supply. Subsequently, a DC–AC inverter transforms the DC power into an AC signal tuned to the system’s resonant frequency. This AC signal excites the primary compensation network and transmitting coil to achieve resonance, thereby enabling efficient energy transfer to the secondary side. On the receiving side, the secondary coil resonates with its own compensation network, and the induced AC power is then rectified by an AC–DC converter to supply DC power to the load.

**Fig 1:**
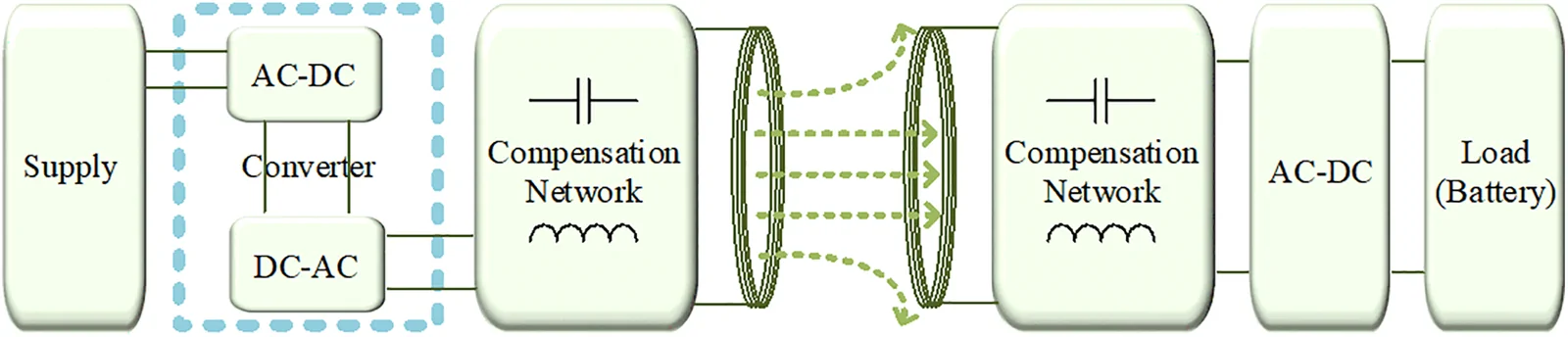
Schematic of the two-coil MCRWPT system.

For analytical purposes, the LCC-S compensated MCRWPT system in Fig 1 can be simplified to the equivalent circuit shown in Fig 2. The input voltage *u*_in_ in Fig 2 represents the square-wave output voltage generated by the high-frequency inverter. In the impedance-based analytical derivation, the resonant-network response is mainly governed by the fundamental-frequency component of this square-wave excitation, because the LCC-S compensation network suppresses most high-order harmonics. In the time-domain Simulink evaluation, the complete square-wave voltage is retained to evaluate the switching behavior of the system. In this model, $u_{\text{in}}$, $i_{\text{in}}$, $i_{\text{1}}$, and $i_{\text{2}}$ denote the primary-side input voltage, primary input current, primary output current, and secondary output current, respectively, with RMS values represented by $U_{\text{in}}$, $I_{\text{in}}$, $I_{\text{1}}$, and $I_{\text{2}}$. $L_{P}$, $L_{\text{1}}$, $L_{\text{2}}$, $C_{P}$, $C_{\text{1}}$, and $C_{\text{2}}$ represent the primary compensation inductance, primary coil self-inductance, secondary coil self-inductance, primary compensation capacitance, primary series capacitance, and secondary series capacitance, respectively. Similarly, $R_{P}$, $R_{\text{1}}$, $R_{\text{2}}$, and $R_{L}$ correspond to the internal resistances of the primary compensation inductor, primary coil, secondary coil, and equivalent load resistance. M represents the mutual inductance between the two coils.

**Fig 2:**
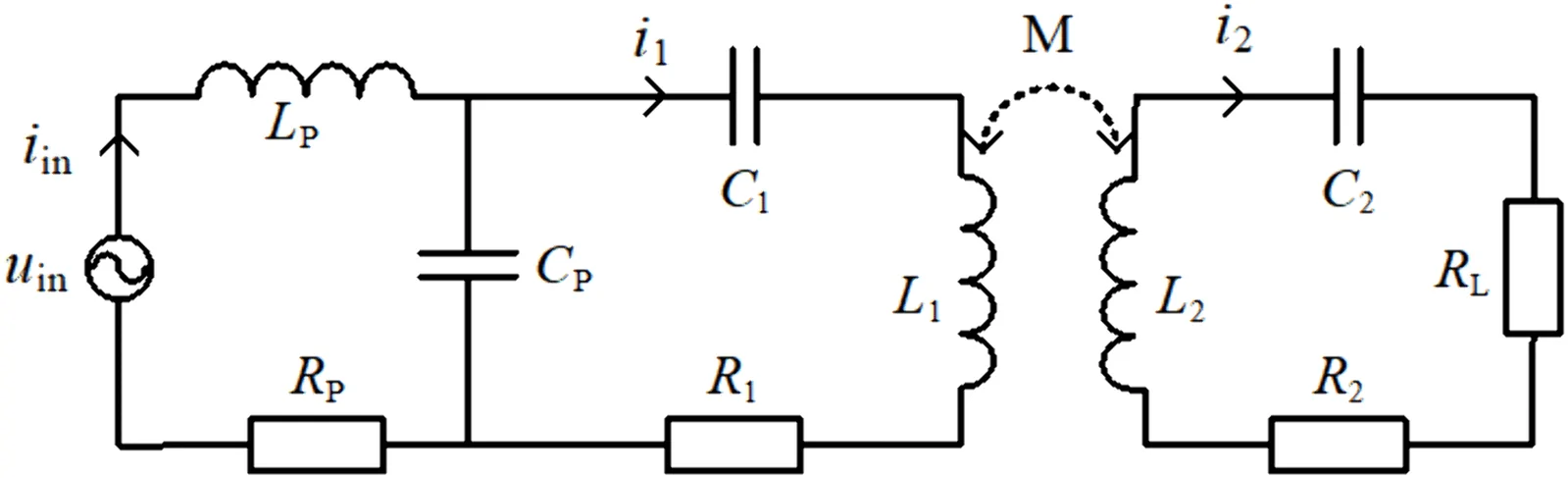
Equivalent circuit of the LCC-S compensated MCRWPT system with inverter square-wave excitation.

Based on the equivalent circuit model shown in Fig 2, the system input impedance $Z_{\text{in}}$ and secondary-side impedance $Z_{\text{2}}$ can be expressed as:


$$\begin{array}{c} \left\{ \begin{array}{c} @l@Z_{\text{in}}=j\omega L_{P}+R_{P}+\frac{\frac{\text{1}}{j\omega C_{P}}\left(j\omega L_{\text{1}}+R_{\text{1}}+Z_{R}+\frac{\text{1}}{j\omega C_{\text{1}}}\right)}{\frac{\text{1}}{j\omega C_{P}}+j\omega L_{\text{1}}+R_{\text{1}}+Z_{R}+\frac{\text{1}}{j\omega C_{\text{1}}}}\\ Z_{\text{2}}=j\omega L_{\text{2}}+\frac{\text{1}}{j\omega C_{\text{2}}}+R_{\text{2}}+R_{L} \end{array}\right. \end{array}$$ (1)

where $Z_{R}$ denotes the reflected impedance from the secondary side to the primary side, expressed as:


$$\begin{array}{c} Z_{R}=\frac{{\left(\omega M\right)}^{\text{2}}}{Z_{\text{2}}} \end{array}$$ (2)

The corresponding branch currents can be written as:


$$\begin{array}{c} \left\{ \begin{array}{c} @l@{\dot{I}}_{\text{in}}=\frac{{\dot{U}}_{\text{in}}}{Z_{\text{in}}}\\ {\dot{I}}_{\text{1}}=\frac{{\dot{I}}_{\text{in}}/\left(j\omega C_{P}\right)}{\frac{\text{1}}{j\omega C_{P}}+\frac{\text{1}}{j\omega C_{\text{1}}}+j\omega L_{\text{1}}+R_{\text{1}}+Z_{R}}\\ {\dot{I}}_{\text{2}}=\frac{j\omega M{\dot{I}}_{\text{1}}}{Z_{\text{2}}} \end{array}\right. \end{array}$$ (3)

When the system operates under full resonance, the transmission performance reaches its optimum. In this state, the system parameters satisfy the following resonance conditions:


$$\begin{array}{c} \left\{ \begin{array}{c} @l@\omega L_{\text{1}}-\frac{\text{1}}{\omega C_{\text{1}}}=\frac{\text{1}}{\omega C_{P}}\\ \omega L_{P}=\frac{\text{1}}{\omega C_{P}}\\ \omega L_{\text{2}}=\frac{\text{1}}{\omega C_{\text{2}}}\\ \omega =\omega_{\text{0}} \end{array}\right. \end{array}$$ (4)

where ω is the operating angular frequency of the system, and $\omega_{\text{0}}$ represents the natural resonant angular frequency. All optimization analyses in this study are performed under the condition of full resonance operation.

According to Eqs. (1)– (4), the output power and transmission efficiency of the system under full resonance can be expressed as:


$$\begin{array}{c} \left\{ \begin{array}{c} @l@P_{\text{out}}=I_{\text{2}}^{\text{2}}R_{L}=\frac{\omega_{\text{0}}^{\text{4}}L_{P}^{\text{2}}M^{\text{2}}R_{L}U_{\text{in}}^{\text{2}}}{Z_{\text{in}}^{\text{2}}(R_{\text{2}}+R_{L}{)}^{\text{2}}(R_{\text{1}}+Z_{R}{)}^{\text{2}}}\mathrm{\backslash vspace}1.5mm\\ \eta =\frac{P_{\text{out}}}{P_{\text{in}}}=\frac{\omega_{\text{0}}^{\text{4}}L_{P}^{\text{2}}M^{\text{2}}R_{L}}{Z_{\text{in}}(R_{\text{2}}+R_{L}{)}^{\text{2}}(R_{\text{1}}+Z_{R}{)}^{\text{2}}}\times 100\% \end{array}\right. \end{array}$$ (5)

The impedance, current, output-power, and efficiency expressions in Eqs. (1)–(5) follow the conventional mutual-inductance equivalent-circuit modeling approach commonly used in resonant and compensated WPT systems [25–28]. These equations are not proposed as new theoretical derivations in this work; instead, they are used to establish the analytical basis and unified notation for the subsequent transmission-characteristic analysis and multi-objective optimization model.

### 2.2. Transmission characteristic analysis

Based on the above theoretical model and following the SAE-J2954 standard for wireless charging of electric vehicles, the system resonant frequency is set to $f_{\text{0}}=85\text{kHz}$, satisfying $\omega_{\text{0}}=2\pi f_{\text{0}}$.

It should be noted that Table 2 lists the reference circuit and simulation parameters of the LCC-S compensated WPT system, whereas not all parameters are treated as independent optimization variables. In this work, the primary compensation inductance *L*_*p*_, equivalent load resistance *R*_*L*_, and mutual inductance *M* are selected as the optimization variables because they represent the compensation-network design parameter, load condition, and magnetic coupling condition, respectively.

**Table 2:** Reference circuit and simulation parameters of the LCC-S compensated WPT system.

Parameter	Value	Parameter	Value	Parameter	Value
*R*_*P*_	0.15 Ω	*U*_in_	400 V	*L*_*P*_	40 μH
*R*_1_	0.15 Ω	*M*	11.75 μH	*L*_1_	70.31 μH
*R*_2_	0.15 Ω	*R*_*L*_	3.6 Ω	*L*_2_	71.56 μH

The self-inductances of the transmitting and receiving coils, *L*_1_ and *L*_2_, are mainly determined by the coil geometry, number of turns, and winding structure. Therefore, they are regarded as fixed hardware parameters in this study. Similarly, the parasitic resistances *R*_*p*_, *R*_1_, and *R*_2_ are mainly determined by the conductor material, winding resistance, and equivalent series resistance of passive components, and thus are not considered as actively adjustable design variables. The compensation capacitors *C*_*p*_, *C*_1_, and *C*_2_ are calculated according to the resonance conditions in Eq. (4) to maintain full-resonance operation and are therefore not independently optimized.

Although the mutual inductance *M* is determined by the coil geometry and relative position, *L*_1_ and *L*_2_ are treated as fixed hardware parameters in the present model. Therefore, varying *M* is used to represent changes in the magnetic coupling condition caused by variations in coil spacing and lateral or angular misalignment. According to $M=k\sqrt{L_{\text{1}}L_{\text{2}}}$, the adopted range of *M* = 1–60 μH, together with *L*_1_ = 70.31 μH and *L*_2_ = 71.56 μH, corresponds approximately to *k* = 0.014–0.846. This intentionally broad interval spans weakly coupled conditions associated with substantial separation or misalignment and strongly coupled conditions associated with close and well-aligned coils. It is used as a prescribed model-based design domain rather than as a universal coupling range for a specific physical coupler. In an actual prototype, the achievable range of *M* would be determined by the coil geometry, air gap, alignment tolerance, shielding structure, and other implementation-specific electromagnetic constraints [27,29].

The bounds in Table 3 define a model-based design space rather than fixed ratings for a particular hardware prototype. The range *L*_*p*_ = 10–40μH keeps the primary compensation inductance below the fixed transmitting-coil self-inductance *L*_1_ = 70.31μH, so that the compensation capacitances determined from the resonance relations in Eq. (4) remain physically meaningful and positive throughout the search domain. At 85 kHz, this interval corresponds to approximately *C*_*p*_ = 350.6–87.6 *nF* and *C*_1_ = 58.1–115.7 *nF*, which provides a practical range for model-level compensation-network adjustment. The load range *R*_*L*_ = 0.1–50 Ω was selected to cover a broad set of equivalent load conditions rather than a single fixed battery-side operating point. *R*_L_ is an equivalent AC-side load used in the circuit model rather than the resistance of a specific physical battery or DC load. The broad range of 0.1–50 Ω was therefore used to examine how the resonant network responds to different reflected/equivalent loading conditions rather than to prescribe a direct hardware load-resistance range. Finally, *M* = 1–60μH represents weak-to-strong magnetic coupling as discussed above. Consequently, the resulting Pareto solutions should be interpreted as candidate combinations within the prescribed design domain; a particular hardware implementation would normally impose narrower ranges according to component ratings, coupler geometry, air gap, and alignment requirements [16,27,29].

**Table 3:** Optimization variables and corresponding ranges.

Variable	Reference value	Optimization range	Physical meaning
*L*_*p*_	40 μH	10–40 μH	Primary compensation inductance
*R*_*L*_	3.6 Ω	0.1–50 Ω	Equivalent load resistance
*M*	11.75 μH	1–60 μH	Mutual inductance/ coupling condition

The MATLAB/Simulink model follows the LCC-S equivalent circuit shown in Fig 2. The reference circuit parameters are listed in Table 2 [30], while the three optimization variables and their bounds are given in Table 3. For each candidate combination of *L*_*p*_, *R*_*L*_, and *M*, the compensation capacitances *C*_*p*_, *C*_1_, and *C*_2_ are recalculated from the resonance conditions in Eq. (4). Using this model, we then evaluated how variations in *L*_*p*_, *R*_*L*_, and *M* affect the output power *P*_out_ and transmission efficiency *η*.

The simulation results are presented in Fig 3. It can be observed that both $P_{\text{out}}$ and η vary significantly with these parameters, reflecting their critical role in determining the overall performance of the LCC-S type MCRWPT system.

**Fig 3:**
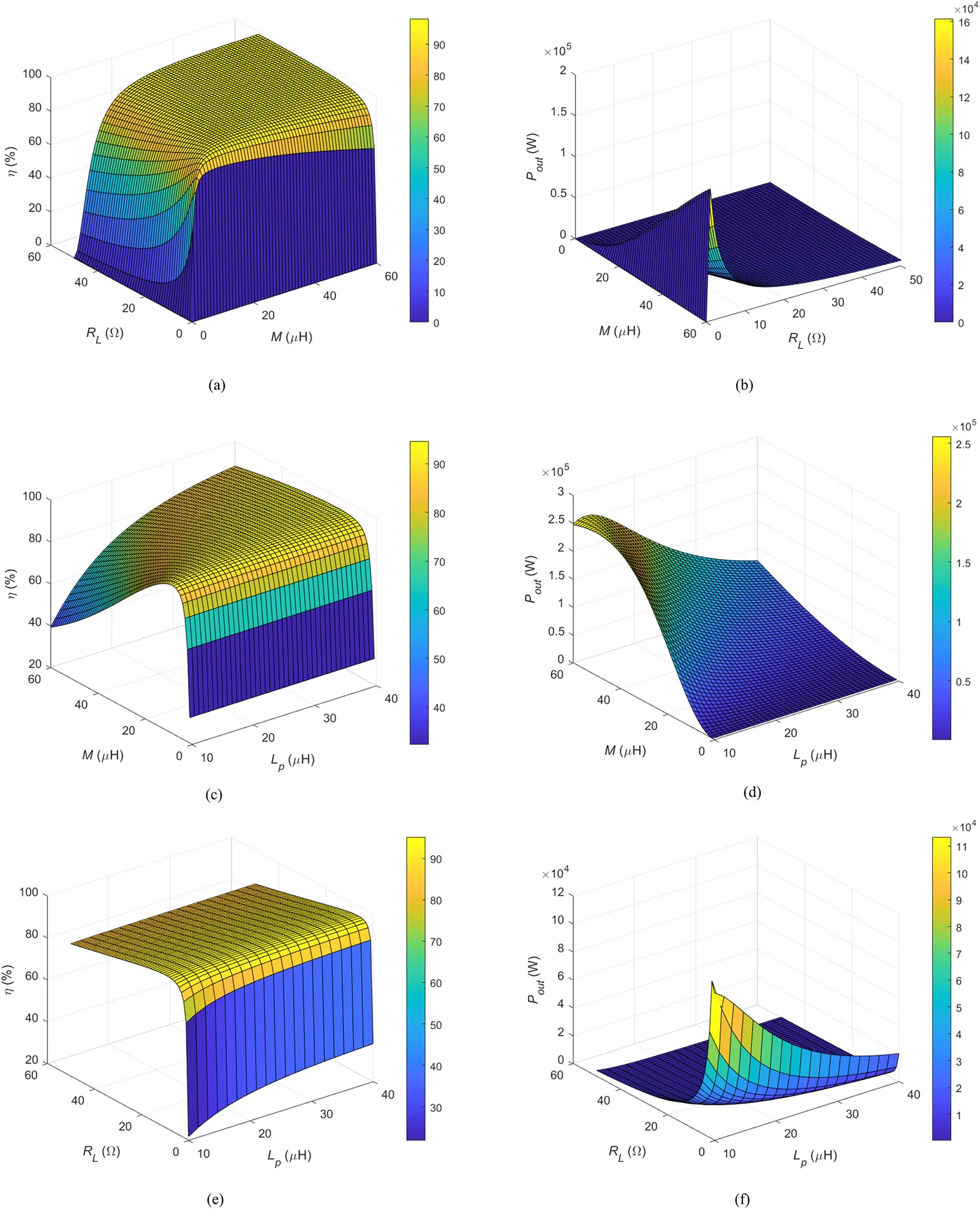
Simulation results showing the influence of compensation inductance $L_{P}$, load resistance $R_{L}$, and mutual inductance M on system output power $P_{out}$ and transmission efficiency η.

Fig 3 shows that the three variables do not act independently. In Fig 3(a), efficiency increases rapidly with *M* in the lower-coupling region and changes more slowly as *M* increases further. The effect of *R*_*L*_ is also non-monotonic, with a relatively favorable load range. Fig 3(b) shows a similar saturation tendency for output power as *M* increases, while *R*_L_ produces a distinct high-power region rather than a monotonic increase. In Figs 3(c) and 3(d), the ridge in the output-power surface indicates that high power is obtained only for particular combinations of *M* and *L*_*p*_. Figs 3(e) and 3(f) show a similar dependence on the joint selection of *L*_*p*_ and *R*_*L*_. These observations support optimizing *L*_*p*_, *M*, and *R*_*L*_ together rather than adjusting them independently.

Output power and transmission efficiency also separate in the Pareto region. Moving toward higher output power generally requires a larger transfer current, stronger coupling, or a load condition closer to the maximum-power region. These conditions increase the modeled loss. Higher efficiency, in contrast, favors lower current stress and lower loss, which limits the achievable output power. We therefore treat $P_{\text{out}}$ and η as conflicting objectives when constructing the Pareto front.

Building on the above analysis, this study formulates a dual-objective optimization problem, with the maximization of transmission efficiency (η) and output power ($P_{\text{out}}$) as the primary goals. The decision variables are selected as $L_{P}$, $R_{L}$, and M. Accordingly, the multi-objective optimization model can be expressed as:


$$\begin{array}{c} max\;F=\left[\begin{array}{c} @c@\eta \left(L_{P},M,R_{L}\right)\\ P_{\text{out}}\left(L_{P},M,R_{L}\right) \end{array}\right] \end{array}$$ (6)

$$\begin{array}{c} s.t.\left\{ \begin{array}{c} @l@L_{P,min}\leq L_{P}\leq L_{P,max}\\ R_{L,min}\leq R_{L}\leq R_{L,max}\\ M_{min}\leq M\leq M_{max}\\ \omega L_{\text{1}}-\frac{\text{1}}{\omega C_{\text{1}}}=\frac{\text{1}}{\omega C_{P}}\\ \omega L_{P}=\frac{\text{1}}{\omega C_{P}}\\ \omega L_{\text{2}}=\frac{\text{1}}{\omega C_{\text{2}}}\\ \omega =\omega_{\text{0}} \end{array}\right. \end{array}$$ (7)

The constraints in Eq. (7) define the prescribed model-based design domain of the optimization problem. The bounds were selected according to the reference circuit parameters, compensation-component feasibility, broad equivalent-load variation, and weak-to-strong magnetic coupling conditions discussed in Section 2.2. They are intended to restrict the numerical search to a physically interpretable design region rather than to define universal hardware limits. In a specific implementation, these bounds should be further restricted according to component ratings, coil geometry, spacing, alignment tolerance, and thermal and electromagnetic constraints. In addition, the resonance conditions in Eq. (4) are imposed throughout the optimization process, and the compensation capacitors are calculated accordingly. Therefore, $C_{P}$, $C_{\text{1}}$, and $C_{\text{2}}$ are treated as dependent parameters rather than independent optimization variables. Based on this formulation, the next section will introduce multi-objective optimization strategies specifically designed for this model, aiming to efficiently solve the non-convex, multi-variable problem and achieve a high-quality configuration for system performance.

## 3. System parameter optimization design

VPSR-PSO uses MOPSO as the multi-objective framework and introduces three search controls: nonlinear dynamic inertia adjustment, probabilistic velocity pausing, and stagnation reset. These mechanisms are used to modify different stages of the search process while retaining the external Pareto archive of MOPSO.

The three mechanisms were selected because *L*_*p*_, *M*, and *R*_*L*_ form a strongly coupled search space. Dynamic inertia changes the search from broader exploration in the early iterations to local refinement later. Velocity pausing limits repeated changes in search direction, while stagnation reset reinitializes particles that stop improving for a specified number of iterations. These mechanisms are not presented as new general-purpose optimization operators. The contribution of the present method lies in their coordinated integration within an archive-based LCC-S Pareto parameter-design procedure and in the quantitative evaluation of their individual and combined roles in this application-specific optimization problem.

### 3.1. Standard PSO algorithm

In the standard PSO algorithm, a number of particles are initialized to search within a defined domain D, where each particle represents a potential solution to the optimization problem. Each particle has two attributes—position and velocity, which correspond to the current solution and the rate of change used to update the position in the next iteration, respectively, and can be expressed as follows:


$$\begin{array}{c} \left\{ \begin{array}{c} @l@\begin{array}{c} @lx_{i}\left(t\right)=\left[x_{i1}\left(t\right),x_{i2}\left(t\right),x_{i3}\left(t\right)\ldots x_{id}\left(t\right)\right] \end{array}\\ v_{i}\left(t\right)=\left[v_{i1}\left(t\right),v_{i2}\left(t\right),v_{i3}\left(t\right)\ldots v_{id}\left(t\right)\right] \end{array}\right. \end{array}$$ (8)

where $x_{i}\left(t\right)$ denotes the position of the *i*-th particle at iteration *t*, d is the dimensionality of the problem, and $v_{i}\left(t\right)$ deno*t*es the velocity of the *i*-th particle at iteration *t*.

In the optimization process, the algorithm updates particle positions by moving them through the search space and tracking two best positions: the personal best and the global best. The personal best represents the optimal solution found by an individual particle, while the global best corresponds to the optimal solution discovered by the entire swarm. Let $pB_{i}$ denote the personal best position of the *i*-th particle and gB denote the global best position of the swarm. The velocity of each particle is updated as follows:


$$\begin{array}{c} v_{i}\left(t+1\right)=wv_{i}\left(t\right)+c_{\text{1}}r_{\text{1}}\left[pB_{i}\left(t\right)-x_{i}\left(t\right)\right]+c_{\text{2}}r_{\text{2}}\left[gB\left(t\right)-x_{i}\left(t\right)\right] \end{array}$$ (9)

where w is the inertia weight, which controls the momentum of particle movement. A larger value of w enhances the global exploration ability of the swarm, while a smaller value promotes local exploitation. $c_{\text{1}}$ and $c_{\text{2}}$ are the acceleration coefficients, representing the cognitive (individual) and social (group) learning factors, respectively. $r_{\text{1}}$ and $r_{\text{2}}$ are random numbers uniformly distributed in [0,1], which introduce stochasticity into the search process, enabling particles to perform random exploration. In the standard PSO algorithm, the inertia weight w is constant. However, in practice, it should be set larger at the beginning of the search to enhance global exploration, and gradually decreased later to strengthen local exploitation. In addition, since each particle updates its velocity at every iteration, it may oscillate between two good solutions or accelerate excessively toward an optimum, thereby missing potential solutions in intermediate regions [31].

Subsequently, the particle position is updated according to the following equation:


$$\begin{array}{c} x_{i}\left(t+1\right)=x_{i}\left(t\right)+v_{i}\left(t+1\right) \end{array}$$ (10)

Following the update of particle velocity and position, the individual best $pB_{i}$ is calculated as follows:


$$\begin{array}{c} pB_{i}\left(t+1\right)=\left\{ \begin{array}{c} @l@x_{i}\left(t+1\right)\;if\;f\left(x_{i}\left(t+1\right)\right)<f\left(pB_{i}\left(t\right)\right)\\ pB_{i}\left(t\right)\;otherwise \end{array}\right. \end{array}$$ (11)

where f(•) denotes the scalar objective function in the conventional single-objective minimization form of standard PSO. In this case, a smaller value of f(•) indicates a better solution, and the personal best position is updated when the current particle position gives a smaller objective value than the previous personal best. The next step is to update the global best position:


$$\begin{array}{c} gB\left(t+1\right)=\left\{ \begin{array}{c} @l@pB_{i}\left(t+1\right)\;if\;f\left(pB_{i}\left(t+1\right)\right)<f\left(gB\left(t\right)\right)\\ gB\left(t\right)\;otherwise \end{array}\right. \end{array}$$ (12)

Eqs. (11) and (12) describe the conventional single-objective PSO update and are included only to define the baseline algorithm. The present problem is different because both *η* and *P*_out_ are maximized. In VPSR-PSO, candidate solutions are therefore compared by Pareto dominance instead of by a single scalar objective. For two candidates *x*_a_ and *x*_b_, *x*_a_ dominates *x*_b_ when *η*(*x*_a_) ≥ *η*(*x*_b_) and *P*_out_(*x*_a_) ≥ *P*_out_(*x*_b_), with at least one strict inequality.

In the following discussion, “objective values” refer to *η* and *P*_out_, whereas the fitness used by the multi-objective search is the corresponding two-dimensional objective vector. No scalar aggregation of the two objectives is used.

### 3.2. VPSR-PSO algorithm

It should be emphasized that, in Section 3.1, the particle position $x_{i}$ denotes a general particle position in the standard PSO framework. Its specific physical meaning depends on the selected design variables of the optimization problem. For the VPSR-PSO implementation in this work, $x_{i}$ is specifically defined as a parameter vector composed of the three selected optimization variables. In the proposed VPSR-PSO implementation, each particle represents a complete candidate parameter configuration of the LCC-S WPT system rather than a single circuit variable. Specifically, the position vector of the *i*-th particle is defined as $x_{i}$ = [$L_{P,i},M_{i},R_{L,i}$], where $L_{P,i}$, $M_{i}$, and $R_{L,i}$ denote the primary compensation inductance, mutual inductance, and equivalent load resistance encoded by this particle, respectively. The corresponding velocity vector is defined as $v_{i}$ = [$v_{L_{P}},v_{M},v_{R_{L}}$]. Therefore, each particle updates the three design variables $L_{P}$, M, and $R_{L}$ simultaneously within the bounds defined in Eq. (7). The resulting candidate parameter combination is evaluated using the objective vector [*η*, *P*_out_].

From Equation (9), it can be observed that the inertia weight w controls the influence of the particle’s previous velocity on its current update speed, which is crucial for balancing the global and local search abilities of the algorithm. In this study, the use of fixed or linearly varying inertia weights is abandoned, and a nonlinear dynamic decay strategy is adopted in combination with the approach proposed in [22]:


$$\begin{array}{c} w\left(t\right)=e^{-(bt/T{)}^{b}} \end{array}$$ (13)

where T denotes the total number of iterations, and b is a constant, typically set to 2.5. The inertia weight determines how strongly the previous particle velocity contributes to the next update. In this study, the fixed inertia weight is replaced by the nonlinear decay in Eq. (13). The larger value during the early iterations allows wider movement in the search space, whereas the lower value in later iterations favors local refinement. The exponential schedule also avoids abrupt changes between different search stages.

At the same time, it is not necessary to update the particle velocity at every iteration, since excessive velocity updates may cause the particles to fall into local optima or miss potential global solutions [32]. Therefore, a velocity pause strategy is introduced in this paper. In each iteration, the velocity of each particle is updated probabilistically according to the following rule:


$$\begin{array}{c} v_{i}\left(t+1\right)=\left\{ \begin{array}{c} @l@v_{i}\left(t\right)\;\;\;if\;rand<\alpha \\ {\left|v_{i}\left(t\right)\right|}^{r_{\text{1}}w\left(t\right)}+c_{\text{1}}r_{\text{1}}\left[pB_{i}\left(t\right)-x_{i}\left(t\right)\right]\\ +c_{\text{2}}r_{\text{2}}\left[gB\left(t\right)-x_{i}\left(t\right)\right]\;otherwise \end{array}\right. \end{array}$$ (14)

where α denotes the velocity pause probability and is defined within the range [0,1]. rand is a random number uniformly distributed in [0,1]. When rand≥α, the particle velocity is updated according to the PSO velocity-update rule. When rand<α, the velocity update is paused, and the particle retains its previous velocity. Therefore, the velocity update probability is 1−α, while the velocity pause probability is α. This probabilistic velocity-pausing strategy prevents particles from changing their search direction too frequently and allows them to further explore promising local regions. In particular, when the particle velocity is relatively small, retaining the previous velocity helps the particle hover around potential Pareto-optimal regions, thereby improving local exploitation and maintaining population diversity. Subsequently, the particle position is updated according to Eq. (10).

In addition, some particles may still fall into local optima and stagnate, which can mislead the entire population into converging to a suboptimal region far from the global optimum. To address this issue, this paper introduces a stagnation reset mechanism. A stagnation counter, ${\text{Stall}}_{i}$, is assigned to each particle and updated according to the following equation:


$$\begin{array}{c} {\text{Stall}}_{i}\left(t+1\right)=\left\{ \begin{array}{c} @l@0\;\;\;\;if\;x_{i}\left(t+1\right)\;dominates\;pB_{i}\left(t\right)\\ {\text{Stall}}_{i}\left(t\right)+1\;otherwise \end{array}\right. \end{array}$$ (15)

Eq. (15) indicates that, during each iteration, if the new position of a particle fails to dominate its individual historical best solution $pB_{i}$, its stagnation counter ${\text{Stall}}_{i}$ is incremented by one. Conversely, if $pB_{i}$ is successfully updated, the counter is reset to zero. The stagnation condition is determined as follows:


$$\begin{array}{c} {\text{Stall}}_{i}\left(t\right)\geq {\text{Stall}}_{max} \end{array}$$ (16)

where ${\text{Stall}}_{max}$ denotes the predefined stagnation threshold. Once this condition is met, the particle undergoes a “reset” operation as defined by the following equation:


$$\begin{array}{c} \left\{ \begin{array}{c} @l@x_{i}\left(t+1\right)=\text{reset}\left(\text{D}\right)\\ v_{i}\left(t+1\right)=0\\ {\text{Stall}}_{i}\left(t+1\right)=0 \end{array}\right. \end{array}$$ (17)

where reset(D) denotes the operation of generating a random position within the search domain D. After the reset, the particle is assigned a completely new position within the entire search space, and both its velocity and stagnation counter are reinitialized.

A stagnation counter records consecutive iterations in which the current particle does not improve its personal best under Pareto dominance. When the counter reaches ${\text{Stall}}_{max}$, the particle is assigned a new random position in the feasible domain, and its velocity and stagnation counter are reset. This step reintroduces search diversity after prolonged stagnation.

The VPSR-PSO algorithm can be applied to single-objective optimization problems; however, in this study, the parameter optimization task is formulated as a multi-objective optimization problem. According to the literature [23], the main distinction between multi-objective and single-objective optimization lies in the introduction of an external archive used to store a set of non-dominated solutions, which collectively form the Pareto front [33]. This set of solutions represents a trade-off between transmission efficiency and output power, providing a compromise set of optimal parameter configurations. Different from the standard single-objective PSO described in Section 3.1, the proposed VPSR-PSO evaluates particles using a multi-objective fitness vector rather than a single scalar fitness value. The fitness vector is defined by the two physical objectives, namely transmission efficiency *η* and output power *P*_out_. Since both objectives are to be maximized, the comparison among particles is conducted according to Pareto dominance. A newly generated solution updates the personal best only when it dominates the previous personal best, while all nondominated solutions are stored and updated in the external Pareto archive.

The optimization process of the VPSR-PSO algorithm proceeds as follows. First, the algorithm initializes all parameters and randomly generates the positions and velocities of the particles. The initial individual best solutions $pB_{i}$ and the initial Pareto archive are then established. During the iterative process, the particle velocities are updated using Eq. (13) and Eq. (14) according to the dynamic inertia weight and velocity pause strategies, while the positions are updated using Equation (10). Instead of using a single global best as in single-objective PSO, each particle selects a leader $g_{i}$ from the Pareto archive to guide its velocity update. The personal best positions are updated according to Eq. (11), and the Pareto archive is continuously refined based on the dominance relationship among solutions. Meanwhile, the stagnation counter of each particle is updated following Eq. (15); once this counter exceeds the threshold defined in Eq. (16), the particle is reinitialized according to Eq. (17). Finally, when the maximum number of iterations is reached, the algorithm outputs the final Pareto-optimal solution set. The Pseudo-code of the VPSR-PSO algorithm is illustrated in Algorithm 1. In Algorithm 1, the personal best is updated according to Pareto dominance, the external archive is maintained by retaining nondominated solutions and removing dominated ones, and the leader used for velocity updating is selected from the current Pareto archive. By combining nonlinear dynamic inertia, velocity pausing, and stagnation reset, VPSR-PSO modifies the search trajectory, velocity-update frequency, and recovery from particle stagnation within the MOPSO framework. The individual contributions of these mechanisms are evaluated separately through the ablation analysis in Section 4.3.

For clarity, the external Pareto archive used by VPSR-PSO accumulates historical nondominated solutions throughout the search. It is initialized from the nondominated feasible solutions in the initial population. After each objective evaluation, a candidate is added to the archive if it is not dominated by any archived solution, while archived solutions dominated by the new candidate are removed. No fixed archive-capacity limit or archive-pruning operation is imposed during the VPSR-PSO search. Because equality of the two objective values is not treated as Pareto dominance, identical objective vectors may temporarily coexist in the search archive. Before the reported performance indicators are calculated, however, the final objective vectors are subjected to a common nondominance filter and duplicate removal. The leader used for particle updating is selected uniformly at random from the current archive.

The computational complexity of VPSR-PSO can be analyzed from Algorithm 1. Let *n* denote the population size, *MaxIt* the maximum number of iterations, *D*_v_ the number of decision variables, *m* the number of objectives, and *A* the size of the external Pareto archive. Position and velocity updating requires O(*D*_v_) operations per particle, whereas dominance checking and archive maintenance may require up to O(*m*A). The resulting search complexity can therefore be expressed approximately as O(*MaxItn*(*D*_v_ + *m*A)). Dynamic-inertia calculation, the velocity-pause decision, and stagnation-counter updating do not change this asymptotic order relative to the underlying MOPSO framework. However, this complexity expression should not be interpreted as implying a general runtime advantage, because practical computation time also depends on archive growth, dominance checking, data structures, implementation details, and the number of stagnation-triggered reset evaluations. The runtime comparisons reported in Section 4.2 therefore refer specifically to the present MATLAB implementation and computing environment.

The specific workflow of the algorithm is illustrated in Fig 4, where the highlighted sections indicate the main improvements introduced in VPSR-PSO. First, the dynamic inertia weight adjustment makes the velocity update more consistent with the characteristics of the optimization process—enabling broader exploration in the early stages and finer exploitation in the later stages. In addition, the velocity pause mechanism effectively balances the exploration and exploitation phases. Meanwhile, the stagnation reset mechanism allows particles to escape from local optima, thereby preventing premature convergence and improving global search capability.

**Fig 4:**
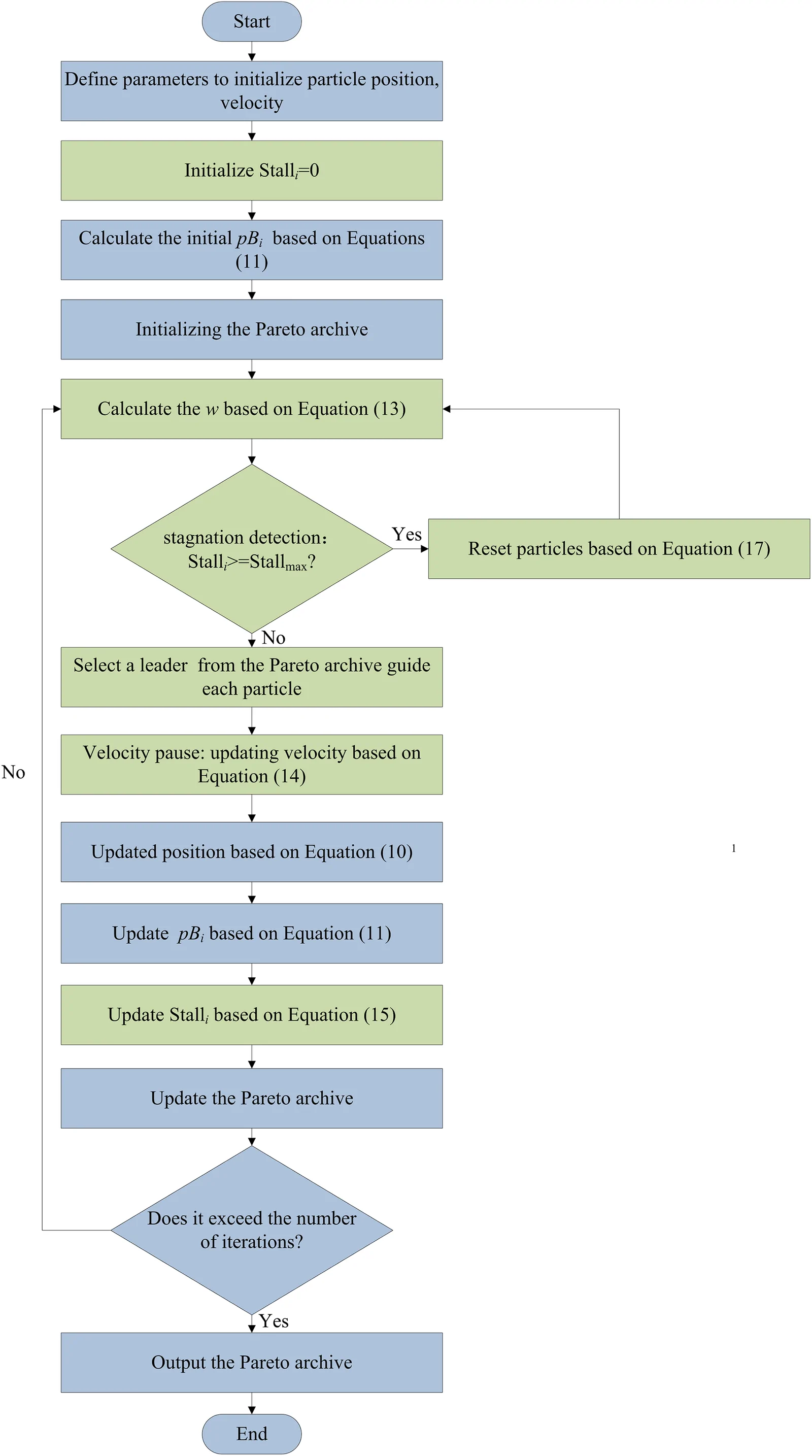
Flowchart of the VPSR-PSO algorithm.

**Algorithm 1. Pseudo-code of VPSR-PSO.**

Define the parameters: population size *n*, number of iterations *MaxIt*, learning factors $c_{\text{1}}$ and $c_{\text{2}}$, pause probability α, and stagnation threshold ${\text{Stall}}_{max}$. Define the optimization function f(•).

**for** *i*=1: *n*

 Randomly initialize particle position $x_{i}$ = [$L_{P,i},M_{i},R_{L,i}$] and set initial velocity $v_{i}$ = 0.

 Evaluate fitness values [$P_{\text{out}i}$, $\eta_{i}$] = $f\left(x_{i}\right)$.

 Set personal best $pB_{i}$ = $x_{i}$.

**end for**

Construct the Pareto archive from all valid solutions.

**for** *t* = 1: *MaxIt*

 Update inertia weight w(t) based on Equation (13).

 **for** *i*=1: *n*

  **if** particle *i* stagnates for more than the threshold based on Equation (16) **then**

   Reinitialize particle *i* based on Equation (17).

  **end if**

  Select a leader $g_{i}$ from the Pareto archive to replace the role of gB in multi-objective optimization framework.

  Generate rand ∈ [0,1];

  **if** rand ≥ α then

   update $v_{i}$ based on Equation (14).

**else**

   Keep the previous velocity unchanged;

**end if**

   Update position $x_{i}$ based on Equation (10).

   Evaluate new objectives [$P_{\text{out}new}$, $\eta_{new}$] = $f\left(x_{i}\right)$.

   **if** [$P_{\text{out}new}$, $\eta_{new}$] dominates $pB_{i}$ **then**

   Update $pB_{i}$ based on Equation (11).

   **else**

   Increase the stagnation counter ${\text{Stall}}_{i}$ based on Equation (15).

   **end if**

   **if** $pB_{i}$ is non-dominated with respect to all solutions in the Pareto archive **then**

    Add $pB_{i}$ to the set and remove all dominated solutions.

   **end if**

  **end for**

**end for**

Output the final Pareto archive A as the obtained Pareto front.

## 4. Simulation evaluation and discussion

This section reports the VPSR-PSO parameter study, benchmark comparison, Pareto-front results, and switching-level evaluation of representative solutions. The analytical LCC-S model is used during optimization, and selected Pareto candidates are subsequently evaluated in MATLAB/Simulink.

### 4.1. Parameter analysis of VPSR-PSO settings

In this subsection, a parametric analysis is conducted to explore the impact of different parameter settings on VPSR-PSO and to guide the parameter configuration for subsequent experiments. The main parameters of the VPSR-PSO algorithm include the particle population size n, individual learning factor $c_{\text{1}}$, population learning factor $c_{\text{2}}$, velocity pause parameter α, and stagnation detection threshold ${\text{Stall}}_{max}$. Among them, the individual and population learning factors have been thoroughly discussed in previous studies [17,18]. Therefore, their values are set according to those references, with both $c_{\text{1}}$ and $c_{\text{2}}$ fixed at 2. The number of algorithm iterations is set to 200. We focus on the effects of the three remaining parameters that are closely related to the proposed algorithm. Parameter-comparison experiments were conducted using the objective function in Equation (6) and the constraints in Equation (7). All experiments were conducted on a computer with an Intel i5 processor, 16 GB of RAM, and MATLAB 2024a. To reduce the influence of stochastic initialization on parameter selection, each candidate setting of population size, velocity-pause probability, and stagnation threshold was evaluated over five independent runs. The same objective model, variable bounds, and computing environment were used in all runs. The quantitative results in Tables 4–6 are therefore reported as mean ± standard deviation over the five runs. Figs 5–7 are retained as representative Pareto-front visualizations, whereas the final parameter choices are based on the repeated-run statistics rather than on a single realization.

**Table 4:** Effect of different population sizes *n* on the algorithm.

*n*	Number of nondominated solutions	HV (normalized)	Spacing (normalized)	Runtime (s)
50	1017.4 ± 70.6	0.7523 ± 0.0021	0.0115 ± 0.0022	7.54 ± 0.64
100	1868.2 ± 64.1	0.7545 ± 0.0012	0.0142 ± 0.0012	25.67 ± 1.36
200	3388.8 ± 66.5	0.7582 ± 0.0007	0.0150 ± 0.0016	82.87 ± 7.67

**Table 5:** Effect of different velocity pause parameter α on the algorithm.

α	Number of nondominated solutions	HV (normalized)	Spacing (normalized)	Runtime (s)
0	5551.2 ± 500.2	0.6734 ± 0.0261	0.0243 ± 0.0016	99.48 ± 15.20
0.3	4681.4 ± 239.5	0.7298 ± 0.0068	0.0335 ± 0.0015	78.45 ± 7.59
0.5	3827.8 ± 193.9	0.7398 ± 0.0068	0.0275 ± 0.0018	59.80 ± 5.36
0.7	1868.2 ± 64.1	0.7582 ± 0.0012	0.0141 ± 0.0012	25.67 ± 1.36

**Table 6:** Effect of different stagnation detection threshold ${Stall}_{max}$ on the algorithm.

${Stall}_{max}$	Number of nondominated solutions	HV (normalized)	Spacing (normalized)	Runtime (s)
5	845.8 ± 24.9	0.7412 ± 0.0030	0.0070 ± 0.0012	7.10 ± 0.66
10	1868.2 ± 64.1	0.7432 ± 0.0012	0.0146 ± 0.0012	25.67 ± 1.36
20	2806.8 ± 146.0	0.7418 ± 0.0031	0.0187 ± 0.0027	104.57 ± 8.12

**Fig 5:**
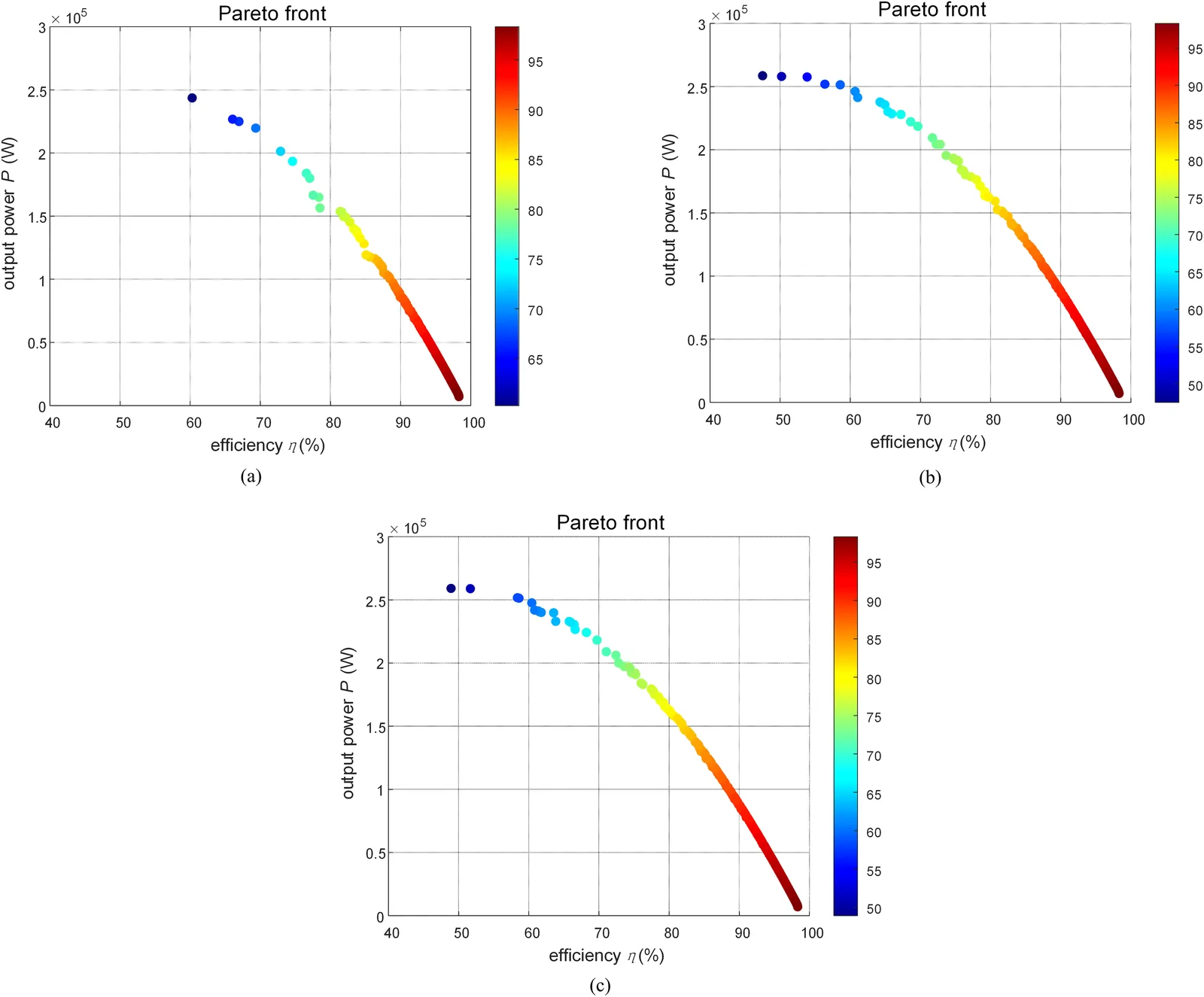
Representative Pareto-front distributions for different population sizes *n* under the same coordinate axes: (a). Pareto frontier distribution for *n* = 50. (b). Pareto frontier distribution for *n* = 100. (c). Pareto frontier distribution for *n* = 200.

**Fig 6:**
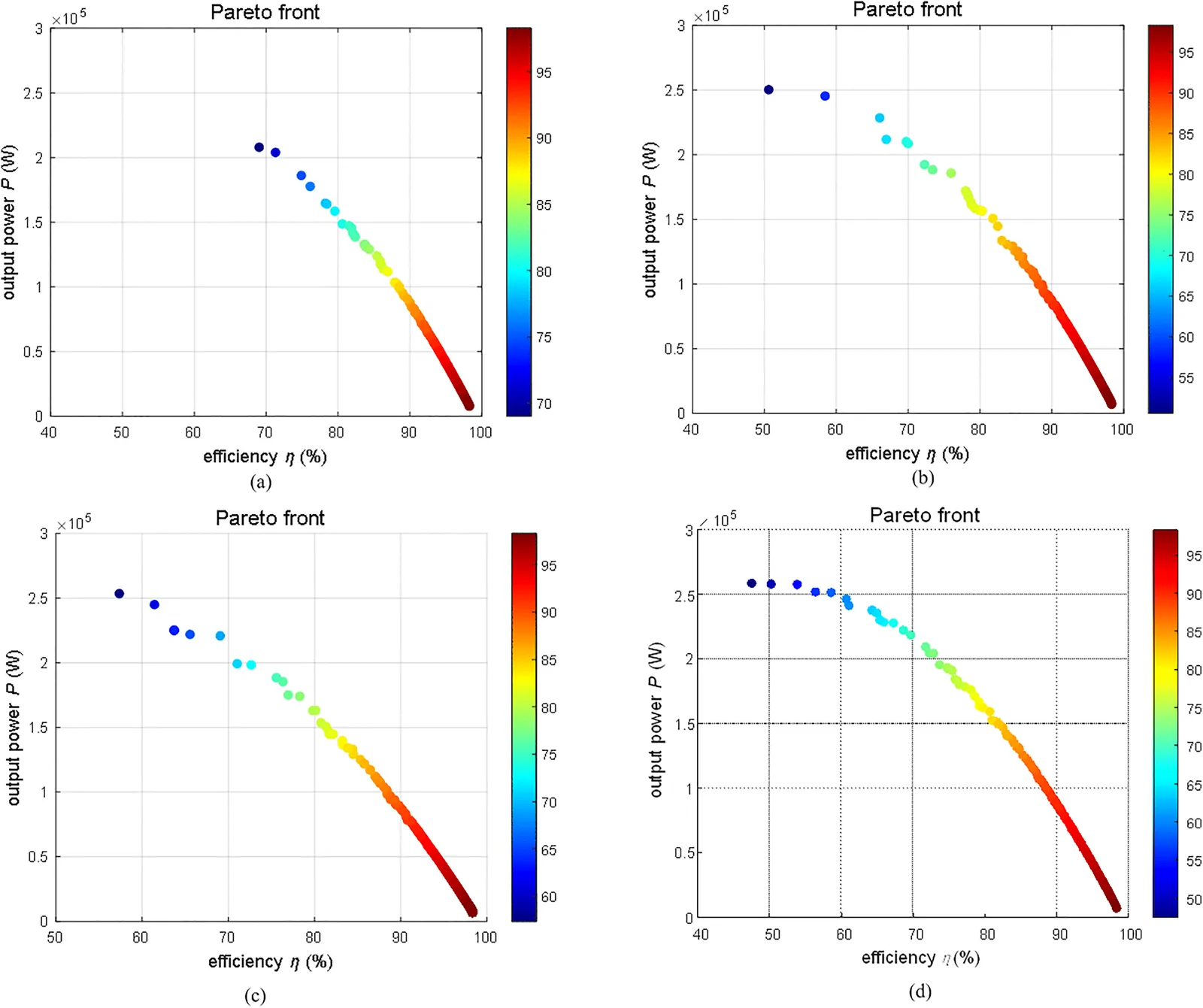
Representative Pareto-front distributions for different velocity pause probabilities α under the same coordinate axes: (a). Pareto frontier distribution for α = 0. (b). Pareto frontier distribution for α = 0.3. (c). Pareto frontier distribution for α = 0.5. (d). Pareto frontier distribution for α = 0.7.

**Fig 7:**
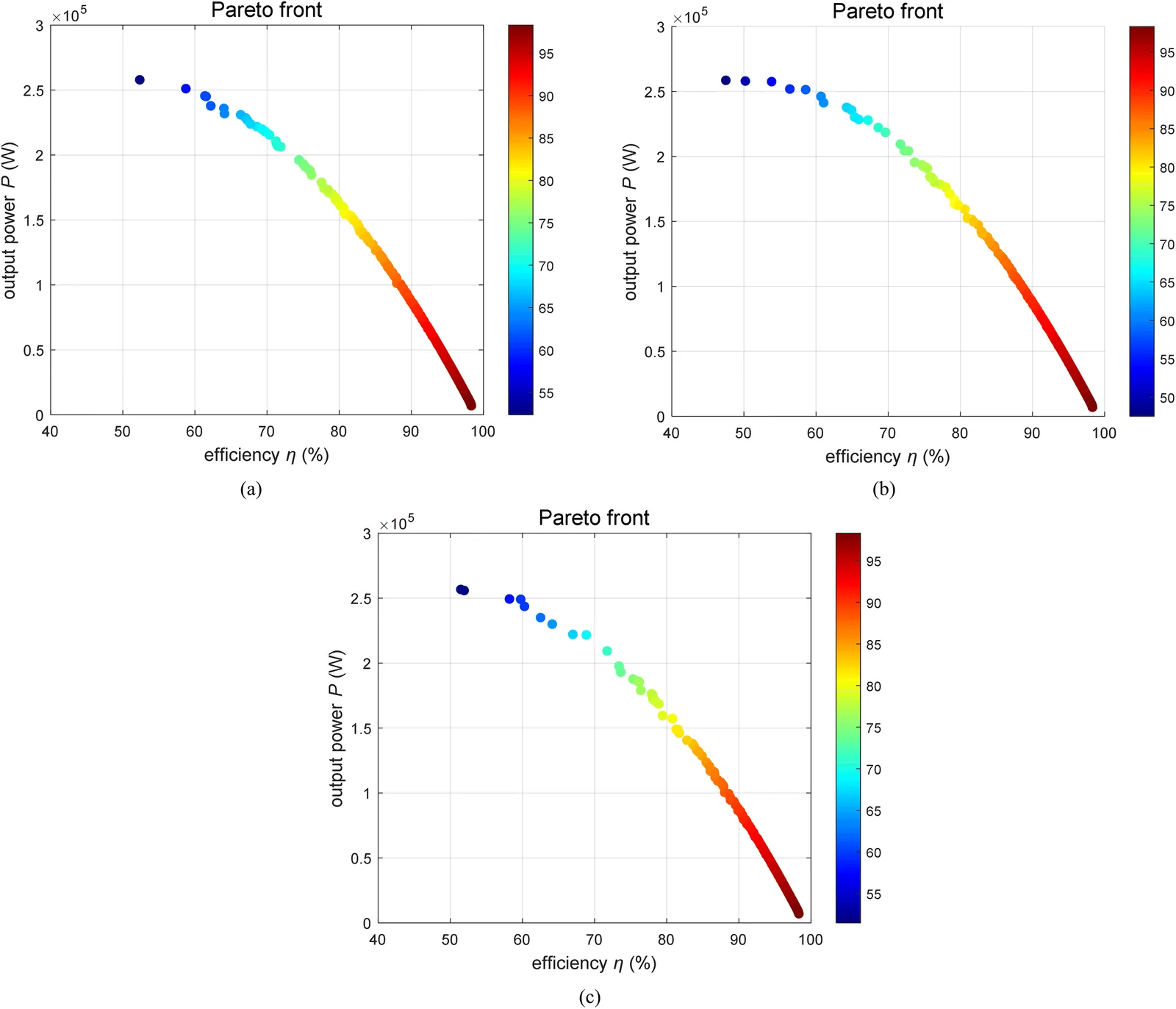
Representative Pareto-front distributions for different stagnation detection thresholds ${Stall}_{max}$ under the same coordinate axes: (a). Pareto frontier distribution for ${\text{Stall}}_{max}$ = 5. (b). Pareto frontier distribution for ${\text{Stall}}_{max}$ = 10. (c). Pareto frontier distribution for ${\text{Stall}}_{max}$ = 20.

To provide a more comprehensive evaluation of the Pareto-front quality under different parameter settings, HV and Spacing are introduced in addition to the number of nondominated solutions and total computation time. The reported solutions refer to the nondominated solutions retained in the external Pareto archive after dominance filtering, while dominated solutions are removed during the archive update process. Therefore, the number of nondominated solutions is used only as an auxiliary indicator. HV is used to evaluate the convergence and coverage of the obtained Pareto front, where a larger value indicates better overall Pareto-front quality. Spacing is used to evaluate the distribution uniformity of the nondominated solutions, where a smaller value indicates a more uniformly distributed Pareto front. Since output power and transmission efficiency have different numerical scales, both objectives are normalized before calculating HV and Spacing. Specifically, within each parameter comparison in this subsection, both objectives were min–max normalized to [0,1] using common bounds determined from all nondominated solutions of the settings included in that comparison. Consequently, the normalized HV and Spacing values are intended for within-table comparison only. Because Tables 4–6 involve different comparison sets and therefore different normalization bounds, the repeated reference configuration n=100, α=0.7, and Stall_max_ = 10 may have different normalized metric values across the three tables. And because both objectives are maximized, HV was calculated in the normalized objective space with the reference point r=(0,0), representing the worst normalized objective vector.

The effect of the particle population size *n* on the algorithm is first discussed. The population size determines how many particles are available to search for the optimal solution during the optimization process, which inevitably affects both the computation time and the quality of the obtained results. Table 4 presents the number of nondominated solutions, HV, Spacing, and the computation time required to find the optimal solutions for *n* = [50, 100, 200], with α = 0.7 and ${\text{Stall}}_{max}$ = 10. The corresponding Pareto-front distributions under the same parameter settings are illustrated in Fig 5.

The representative fronts in Fig 5 illustrate the corresponding distribution patterns, while the repeated-run quantitative results are summarized in Table 4. The repeated-run results in Table 4 show that increasing the population size improves Pareto-front coverage but also increases computational cost substantially. The highest mean HV is obtained with n =200 (0.7582), compared with 0.7545 for n =100; however, this approximately 0.49% HV increase is accompanied by an increase in mean runtime from 25.67 s to 82.87 s. Although n=50 provides the shortest runtime and the lowest mean Spacing, its mean HV is lower and the resulting nondominated set is substantially smaller. Therefore, n=100 is retained as a compromise between Pareto-front quality, solution-set coverage, and observed implementation-level runtime rather than as the setting that optimizes every individual metric.

The velocity pause parameter α plays a crucial role in the VPSR-PSO algorithm. It controls the rate at which particles adjust their velocity during iterations, thereby influencing the balance between local exploitation and global exploration. A smaller α leads to more frequent velocity updates and stronger global exploration, whereas a larger α results in fewer updates and more meticulous searches around promising regions. In the experiments for this parameter, the population size was set to *n* = 100 and ${\text{Stall}}_{max}$ = 10. Table 5 reports the number of nondominated solutions, HV, Spacing, and the computation time when α = [0, 0.3, 0.5, 0.7], while Fig 6 illustrates the corresponding Pareto-front distributions under the same parameter settings. When α = 0, the velocity pause mechanism is disabled, meaning that all particles continuously update their velocities during the iterations.

The repeated-run results provide a consistent preference for α=0.7. Its mean HV of 0.7582 is the highest among the tested settings, while its mean Spacing of 0.0141 and runtime of 25.67 s are the lowest. In addition, the relatively small HV standard deviation indicates stable performance across independent initializations. In contrast, disabling velocity pausing α=0 substantially reduces mean HV and increases both Spacing and runtime. These results indicate that, for the present multi-objective LCC-S problem, a relatively high velocity-pause probability reduces unnecessary changes in particle search direction while the stagnation-reset mechanism maintains exploration when particles cease to improve. Although Reference [22] used α=0.3 for different single-objective test problems, the repeated-run results here favor α=0.7 for the present multi-objective LCC-S design problem. Accordingly, α=0.7 is retained for the subsequent experiments.

The third parameter considered is the stagnation detection threshold, ${\text{Stall}}_{max}$, which determines how many consecutive iterations a particle is allowed to show no improvement before being reset. Experiments were conducted with *n* = 100 and α = 0.7. Table 6 reports the number of nondominated solutions, HV, Spacing and the algorithm runtime under different ${\text{Stall}}_{max}$ settings of [5,10,20]. The corresponding Pareto front distributions are illustrated in Fig 7.

As shown in Fig 7 and Table 6, the stagnation threshold affects the balance between search continuation and random reinitialization. The repeated-run results show that the stagnation threshold controls a clear trade-off between reset frequency, Pareto-front coverage, and computational cost. A threshold of 5 produces the shortest runtime and the lowest mean Spacing, but frequent resets limit historical front coverage and yield the lowest mean HV among the three tested settings. Increasing the threshold to 20 allows substantially more nondominated solutions to accumulate but increases the mean runtime to 104.57 s without improving HV. A threshold of 10 produces the highest mean HV (0.7432) and the smallest HV standard deviation, while maintaining substantially lower runtime than the threshold of 20. Therefore, ${\text{Stall}}_{max}$ =10 is retained as a balanced setting that favors overall Pareto quality and run-to-run stability rather than the minimum Spacing alone.

Based on the repeated-run parameter analysis, *n* = 100, α =0.7, and ${\text{Stall}}_{max}$ =10 are retained for the subsequent benchmark comparison. These values are selected as robust compromise settings across Pareto-front quality, run-to-run variability, solution-set coverage, and computational cost rather than as settings that independently optimize every reported metric.

### 4.2. Benchmark comparison with representative optimization algorithms

To further evaluate the performance of the proposed VPSR-PSO framework, MOPSO [23], NSGA-II [34], and MOGWO [35] were included as representative multi-objective comparison algorithms. NSGA-II represents a population-based evolutionary algorithm, whereas MOGWO provides an additional swarm-intelligence-based comparison. Recent WPT studies have also considered multi-objective optimization. Wu et al. [36] applied NSGA-II to a detuned LCC-S dynamic WPT system under mutual- and self-inductance drift, while Wu et al. [37] combined an adaptive DNN model with improved PSO for multi-objective coil design. More recent studies have further explored multi-objective optimization in LCC-S WPT systems. Liu et al. [38] employed MOPSO for data-efficient multi-objective design of LCC-S auxiliary localization coils under misalignment conditions, whereas Zhao et al. [39] used NSGA-II for LCC-S parameter optimization under coupling and load variations. Because their optimization variables, objectives, and system configurations differ from those considered here, their numerical results are not directly comparable with the present study.

The four algorithms were therefore evaluated under a controlled computational protocol using the same LCC-S analytical model, objective functions, decision-variable bounds, population size, maximum number of iterations, and computing platform. The stochastic guidance rules used in the comparison were also implemented consistently with the random-control versions adopted in this study: VPSR-PSO, MOPSO, and MOGWO select archive leaders randomly, while equal-rank selection in the implemented NSGA-II comparison is resolved randomly. Algorithm-specific search and archive mechanisms were otherwise retained rather than replacing them with a newly imposed common archive structure. The common experimental settings and the VPSR-PSO-specific parameters are summarized in Table 7.

**Table 7:** Parameter settings used in the benchmark comparison.

Parameter	Value	Description
Population size *n*	100	Used for all algorithms
Maximum iterations *MaxIt*	200	Used for all algorithms
Independent runs	10	Used for statistical comparison
Decision variables	$R_{L}$, M, $L_{P}$	Same variable bounds for all algorithms
Objectives	$P_{\text{out}}$, η	Same objective functions for all algorithms
$c_{\text{1}}$	2	VPSR-PSO/ MOPSO acceleration coefficient
$c_{\text{2}}$	2	VPSR-PSO/ MOPSO acceleration coefficient
α	0.7	Velocity pause probability of VPSR-PSO
${\text{Stall}}_{max}$	10	Stagnation threshold of VPSR-PSO

The archive and nondominated-solution recording procedures were explicitly examined because they differ among the implemented algorithms. In VPSR-PSO and MOPSO, the external archive accumulates historical nondominated solutions without a predefined capacity limit. The NSGA-II implementation retains a fixed evolutionary population of 100 individuals and additionally records historical nondominated solutions for subsequent Pareto-front evaluation; duplicate objective vectors are rejected using a numerical tolerance of 10^−10^. In MOGWO, the external archive is limited to 5000 solutions and is randomly truncated only if this limit is exceeded; duplicate objective vectors are likewise rejected using a numerical tolerance of 10^−10^. Thus, the archive-management mechanisms are not identical across the four algorithms, and the benchmark is interpreted as a controlled comparison of the implemented methods rather than as a comparison under an identical archive policy.

To reduce the direct influence of different archive sizes on the reported Pareto-quality indicators, all final solution sets were processed using the same evaluation procedure. Objective values from all compared runs were normalized using a common global min–max range. The resulting objective vectors were subjected to the same nondominance filtering and duplicate removal before metric calculation. HV was calculated from the complete filtered nondominated front, whereas Spacing was evaluated using 500 points uniformly sampled from each normalized Pareto front. The reported number of nondominated solutions therefore refers to the unique solutions remaining after the common final filtering procedure and is treated only as an auxiliary indicator.

With a population size of 100 and 200 iterations, MOPSO, NSGA-II, and MOGWO require 20,100 objective-function evaluations per run, including evaluation of the initial population. VPSR-PSO follows the same nominal population and iteration settings, but a stagnation-triggered reset introduces an additional objective evaluation for the reset particle. Therefore, its actual number of objective-function evaluations is 20,100 plus the number of reset evaluations. The present benchmark is consequently interpreted as a controlled implementation-level comparison rather than an identical function-evaluation-budget comparison.

All four algorithms were implemented in MATLAB R2024a and executed serially on the same Intel i5 computer with 16 GB of RAM. The same analytical LCC-S objective-evaluation function was used for all algorithms, with candidate solutions evaluated individually rather than through algorithm-specific vectorized batch evaluation, and no parallel computation was applied. Runtime was measured using MATLAB’s tic/toc around each optimization run. It therefore includes the algorithmic search operations performed during the run, including objective evaluation, particle or population updating, dominance checking, and archive maintenance. The subsequent cross-algorithm normalization, statistical testing, figure generation, and other reporting-oriented post-processing were not included in the reported runtime. Accordingly, the runtime values should be interpreted as implementation-level measurements under the reported software and hardware conditions rather than as hardware-independent measures of algorithmic complexity.

The comparison was evaluated using HV, Spacing, the number of unique nondominated solutions, and runtime. HV reflects both the convergence and coverage of the obtained Pareto front, with a larger value indicating better overall Pareto-front quality. Spacing reflects the distribution uniformity of the nondominated solutions, with a smaller value indicating a more even distribution. The number of unique nondominated solutions is reported only as an auxiliary indicator because a larger solution count does not necessarily imply better Pareto-front quality. Runtime represents the observed computation time of the corresponding MATLAB implementation under the reported computing environment. To quantify the uncertainty associated with stochastic optimization, 95% confidence intervals were calculated across the 10 independent runs. Because the runs of the different algorithms were independently initialized rather than treated as paired experimental blocks, overall differences among the four algorithms were first assessed using the Kruskal–Wallis test. Pairwise comparisons between VPSR-PSO and each comparison algorithm were subsequently performed using two-sided Wilcoxon rank-sum tests, with Holm correction applied to the resulting p-values to control for multiple comparisons. Cliff’s delta was additionally reported as an effect-size measure. A significance level of 0.05 was used for the statistical tests.

Based on the above benchmark settings, the Pareto-front distributions and convergence behaviors of the compared algorithms are shown in Figs 8–10. For clearer visualization, the Pareto-front points in Fig 8 were uniformly sampled along the output-power axis for each algorithm.

**Fig 8:**
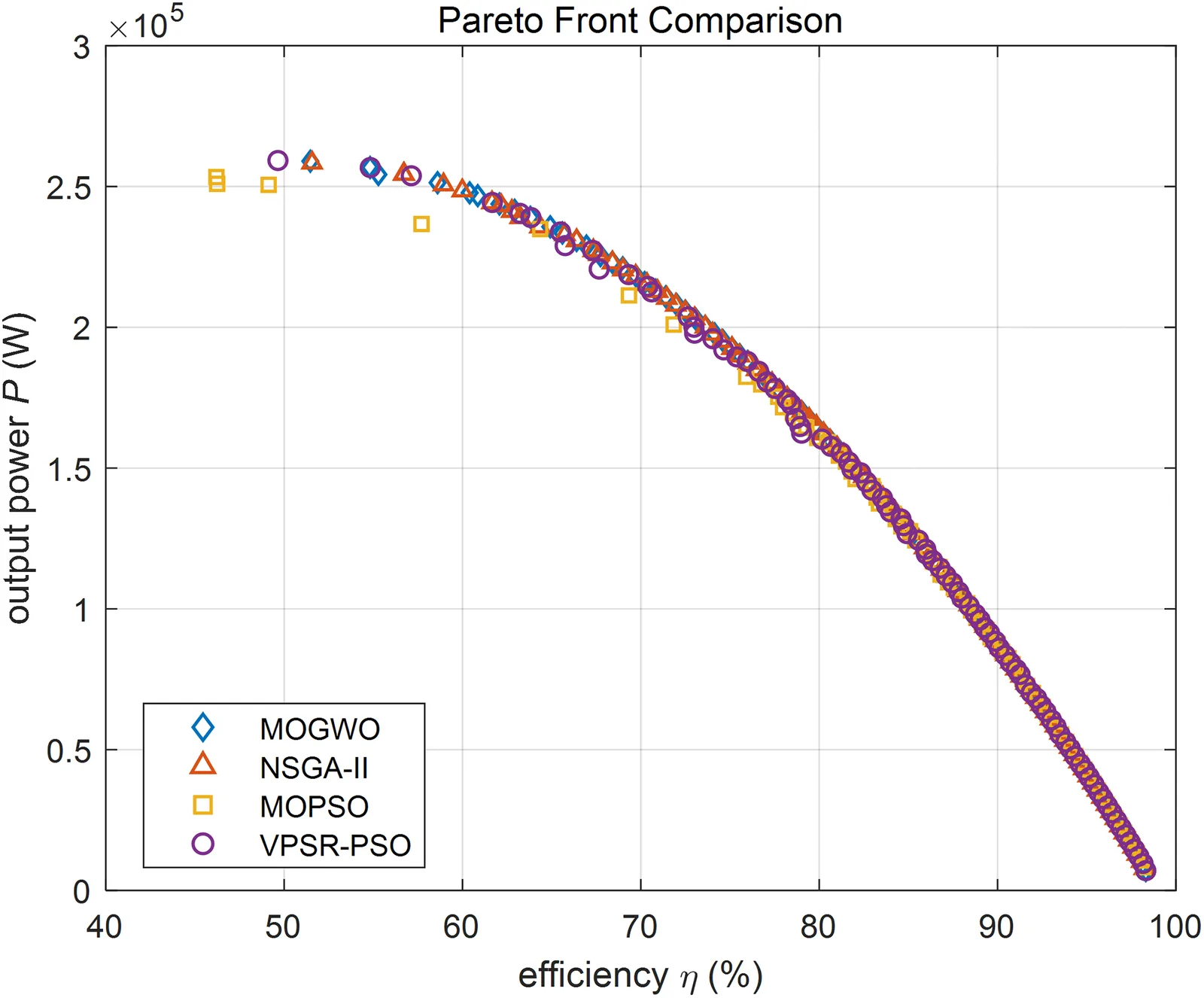
Pareto front comparison of VPSR-PSO, MOPSO, NSGA-II, and MOGWO under the same optimization settings.

**Fig 9:**
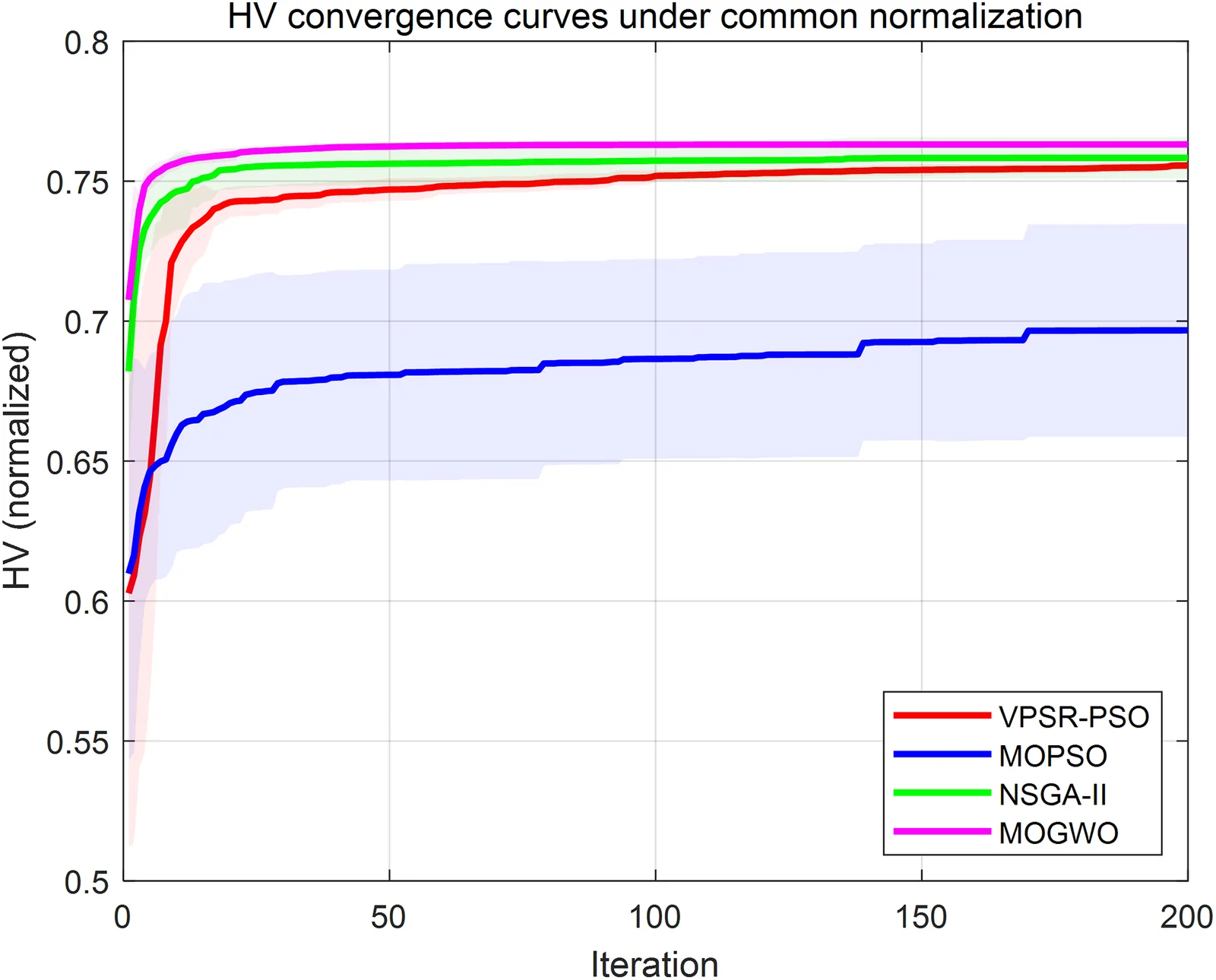
HV convergence curves of the compared algorithms under common normalization over 10 independent runs.

**Fig 10:**
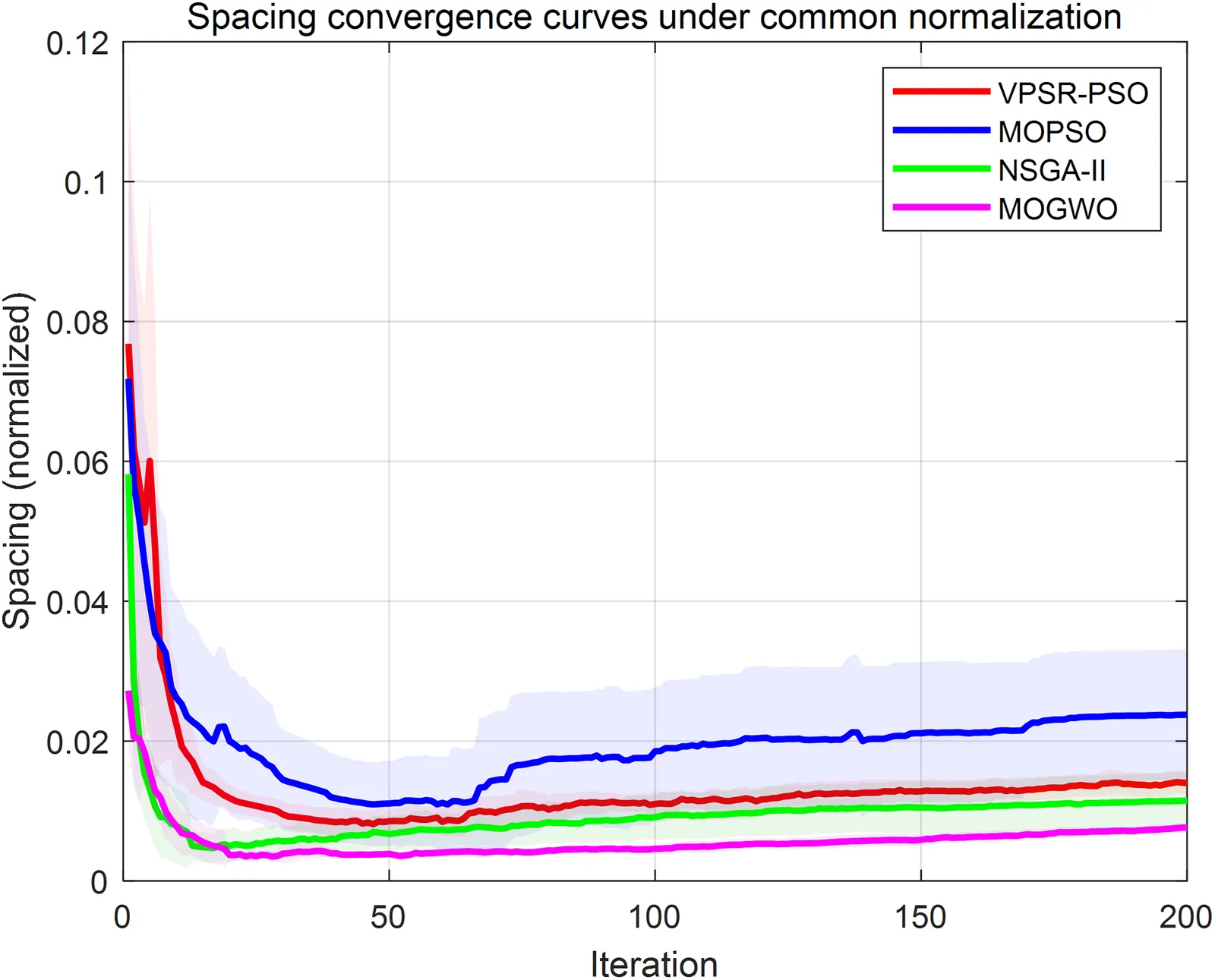
Spacing convergence curves of the compared algorithms under common normalization over 10 independent runs.

Fig 8 compares the final Pareto fronts obtained by the four algorithms. All algorithms capture the main trade-off relationship between output power and transmission efficiency. Compared with MOPSO, VPSR-PSO provides a more stable and better-covered Pareto front in the main trade-off region. This suggests that the proposed improvement strategies help enhance the search capability of the baseline PSO framework under the tested LCC-S parameter-design setting. NSGA-II and MOGWO provide slightly denser Pareto-front distributions, which is reasonable because these algorithms include explicit diversity-preservation mechanisms. Nevertheless, VPSR-PSO still captures the main Pareto-front trend effectively and provides a compact set of representative trade-off solutions.

The HV convergence curves in Fig 9 further illustrate the convergence behavior of the compared algorithms. VPSR-PSO converges rapidly in the early iterations and reaches a higher HV value than MOPSO under the tested setting. Moreover, the fluctuation range of MOPSO is noticeably larger, indicating that the baseline MOPSO is more sensitive to random initialization and may suffer from unstable search behavior. In contrast, VPSR-PSO shows a smoother and more stable convergence process, suggesting that the proposed velocity-pausing and stagnation-reset mechanisms help improve search stability and stagnation avoidance. Although NSGA-II and MOGWO reach higher final HV values, VPSR-PSO remains close to them while showing shorter observed runtimes under the present MATLAB implementation. This indicates that the proposed velocity-pausing and stagnation-reset mechanisms mainly improve the search stability and observed runtime behavior of the PSO-based implementation, rather than simply increasing the number of nondominated solutions.

Fig 10 presents the Spacing convergence curves of the compared algorithms. VPSR-PSO obtains a lower final Spacing value than MOPSO, suggesting better distribution uniformity of the nondominated solutions than the baseline PSO-based method under the tested setting. NSGA-II and MOGWO achieve lower Spacing values, which can be attributed to their explicit diversity-maintenance mechanisms. Therefore, the Spacing results should be interpreted together with HV and runtime: NSGA-II and MOGWO provide stronger distribution uniformity, whereas VPSR-PSO improves the baseline MOPSO and provides a compromise between Pareto-front quality and observed implementation-level runtime under the present experimental conditions. Overall, the comparison indicates that VPSR-PSO improves the baseline MOPSO under the tested settings and provides a practical balance between Pareto-front quality and computational cost compared with NSGA-II and MOGWO.

To further quantify the observations from the Pareto-front and convergence-curve comparisons, the statistical results over 10 independent runs are summarized in Table 8.

The nonparametric analysis confirmed significant overall differences among the four algorithms for both HV (Kruskal–Wallis H=33.596, p=2.41 × 10^−7^) and Spacing (H=30.121, p=1.30 × 10^−6^). In the Holm-corrected pairwise comparisons, VPSR-PSO achieved a significantly higher HV than MOPSO (p=5.48 × 10^−4^, Cliff’s δ=1.00) and a significantly lower Spacing (p=1.54 × 10^−3^, δ =−0.90). In contrast, NSGA-II showed a higher HV than VPSR-PSO (p=7.28 × 10^−3^, δ=−0.72) and a lower Spacing (p=3.76 × 10^−2^, δ=0.56). MOGWO also outperformed VPSR-PSO in HV (p=5.48 × 10^−4^, δ=−1.00) and Spacing (p=5.48 × 10^−4^, δ=1.00). The signs of Cliff’s delta are defined relative to VPSR-PSO, with a positive value indicating larger values for VPSR-PSO. These results statistically support the improvement of VPSR-PSO over the MOPSO baseline, while also confirming that NSGA-II and MOGWO provide better final Pareto-quality indicators under the tested conditions.

The number of nondominated solutions is treated only as an auxiliary indicator because a larger solution set does not necessarily imply better Pareto-front quality when redundant or unevenly distributed solutions are present. Consistent with the statistical tests, Table 8 shows that VPSR-PSO improves the baseline MOPSO in both Pareto quality and observed runtime. Mean HV increases from 0.6967 for MOPSO to 0.7556 for VPSR-PSO, while mean Spacing decreases from 0.0238 to 0.0140 and runtime decreases from 53.49s to 39.42s. In contrast, NSGA-II and MOGWO obtain better final Pareto-quality indicators than VPSR-PSO, but require substantially longer computation times of 302.91s and 87.58s, respectively. Thus, the principal benefit of VPSR-PSO under the present implementation is its improvement over the MOPSO baseline together with a favorable balance between Pareto-front quality and computation time, rather than superiority in every optimization metric.

Compared with NSGA-II, the mean HV of VPSR-PSO is lower by approximately 0.36%, whereas its observed runtime is approximately 86.99% shorter under the present MATLAB implementation. Compared with MOGWO, VPSR-PSO also shows lower HV and higher Spacing, while requiring approximately 54.99% less runtime. These differences indicate that VPSR-PSO does not provide the best final Pareto-quality metrics among all tested algorithms. Instead, it offers an application-specific compromise between solution quality and implementation-level runtime for the LCC-S WPT parameter-design problem.

**Table 8:** Statistical comparison of different multi-objective optimization algorithms over 10 independent runs.

Algorithm	HV (normalized)	Spacing (normalized)	Number of unique nondominated solutions	Runtime (s)
VPSR-PSO	0.7556 ± 0.0014 [0.7546, 0.7566]	0.0140 ± 0.0017 [0.0128, 0.0152]	1861.8 ± 56.0	39.42 ± 1.72 [38.19, 40.65]
MOPSO	0.6967 ± 0.0380 [0.6695, 0.7239]	0.0238 ± 0.0094 [0.0171, 0.0305]	2735.3 ± 205.2	53.49 ± 6.02 [49.18, 57.80]
NSGA-II	0.7583 ± 0.0075 [0.7530, 0.7637]	0.0115 ± 0.0041 [0.0086, 0.0144]	4577.5 ± 231.0	302.91 ± 14.40 [292.61, 313.21]
MOGWO	0.7631 ± 0.0002 [0.7630, 0.7633]	0.0076 ± 0.0007 [0.0071, 0.0082]	4460.7 ± 49.6	87.58 ± 1.43 [86.56, 88.60]

Note: Values are reported as mean ± standard deviation, with 95% confidence intervals shown in square brackets for HV, Spacing, and runtime.

### 4.3. Ablation analysis of VPSR-PSO components

To isolate the contribution of the three search mechanisms incorporated into VPSR-PSO, an ablation analysis was conducted using five configurations: the baseline MOPSO, the complete VPSR-PSO, VPSR-PSO without nonlinear dynamic inertia, VPSR-PSO without velocity pausing, and VPSR-PSO without stagnation reset. In the no-dynamic-inertia variant, the nonlinear inertia schedule in Eq. (13) was disabled and the inertia factor was held at its initial-scale value. In the no-velocity-pause variant, the pause probability was set to zero, so the velocity was updated at every iteration. In the no-stagnation-reset variant, the reset operation in Eqs. (16)–(17) was disabled. Unless otherwise stated, the remaining settings were identical to those used for the complete VPSR-PSO. Each configuration was evaluated over five independent runs. The same normalization and metric-processing procedure was applied to all five configurations within the ablation experiment. The normalized HV and Spacing values in this subsection are therefore intended for comparisons among the ablation configurations and should not be directly compared numerically with those in Table 8, whose normalization range was obtained from the four-algorithm benchmark.

Table 9 shows that the three mechanisms do not contribute to the final performance in the same manner. Removing velocity pausing causes the largest deterioration in Pareto-front quality: the mean HV decreases from 0.7440 to 0.6639, while Spacing increases from 0.0146 to 0.0249 and runtime increases markedly. This result indicates that probabilistic velocity pausing is the main mechanism responsible for suppressing excessive search-direction changes and improving Pareto-front quality and the observed runtime behavior of the PSO-based search under the present implementation. Removing the stagnation-reset mechanism also degrades the results, reducing mean HV to 0.7333 and increasing Spacing to 0.0272. More notably, the mean runtime increases from 25.67 s to 268.75 s, indicating that reinitializing persistently stagnated particles substantially reduces prolonged ineffective search and historical archive accumulation.

**Table 9:** Ablation results of VPSR-PSO components over five independent runs.

Configuration	HV (normalized)	Spacing (normalized)	Runtime (s)	NFE
MOPSO baseline	0.6800 ± 0.0415	0.0263 ± 0.0089	40.97 ± 3.73	20,100
Full VPSR-PSO	0.7440 ± 0.0012	0.0146 ± 0.0012	25.67 ± 1.36	21,932.2 ± 9.3
w/o dynamic inertia	0.7447 ± 0.0027	0.0116 ± 0.0015	25.03 ± 1.66	21,932.6 ± 7.2
w/o velocity pause	0.6639 ± 0.0249	0.0249 ± 0.0016	99.48 ± 15.20	21,963.4 ± 6.1
w/o stagnation reset	0.7333 ± 0.0058	0.0272 ± 0.0044	268.75 ± 10.66	20,100

Note: Values are reported as mean ± standard deviation over five independent runs. Larger HV and smaller Spacing are preferred.

The effect of nonlinear dynamic inertia is more limited and should be interpreted differently. Removing this mechanism produces a slightly higher final mean HV (0.7447 versus 0.7440) and a lower mean Spacing value, while the difference in runtime is small. Therefore, the ablation results do not support attributing an independent improvement in final Pareto-front quality to the dynamic-inertia schedule. However, the complete VPSR-PSO exhibits lower run-to-run variation in the final HV (standard deviation 0.0012 versus 0.0027), and the convergence histories show reduced HV dispersion during much of the search, particularly in the later iterations. The role of dynamic inertia in the present framework is therefore interpreted primarily as contributing to search stability rather than increasing the final mean HV.

Fig 11 confirms that the largest convergence degradation occurs when velocity pausing is removed. The no-stagnation-reset variant can approach the full method during the early search but subsequently exhibits poorer HV and Spacing behavior as stagnated particles accumulate. The dynamic-inertia ablation follows the full method relatively closely and eventually reaches a comparable or slightly higher mean HV; however, Table 9 shows greater run-to-run variability in its final HV than in the complete VPSR-PSO. These observations are consistent with Table 9 and indicate that velocity pausing and stagnation reset provide the main performance gains, whereas nonlinear dynamic inertia mainly affects the stability of the search trajectory.

**Fig 11:**
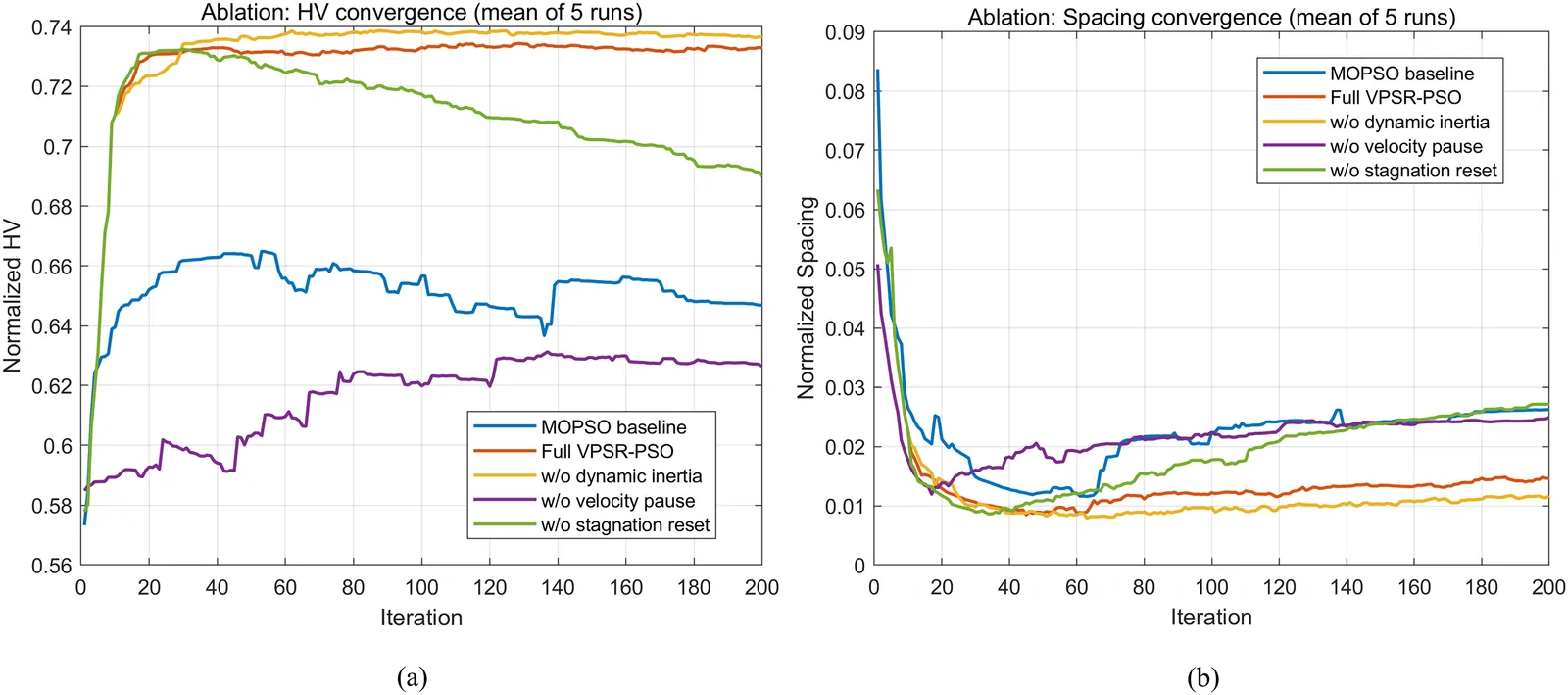
Convergence behavior in the VPSR-PSO ablation study over five independent runs: (a) normalized HV convergence and (b) normalized Spacing convergence. The curves represent the mean values over five independent runs.

### 4.4. Analysis of Pareto-front solutions

Following the comparative analysis, the VPSR-PSO framework was applied to the LCC-S WPT parameter-design problem defined by the objective formulation in Eq. (6) and the constraints in Eq. (7). Transmission efficiency η and output power $P_{\text{out}}$ were treated as the two objectives, with $L_{P}$, $R_{L}$, and M as the decision variables. The parameter settings are provided in Table 7. For the subsequent representative-solution analysis, Pareto candidates satisfying η≥90% were retained, and the resulting front is shown in Fig 12.

**Fig 12:**
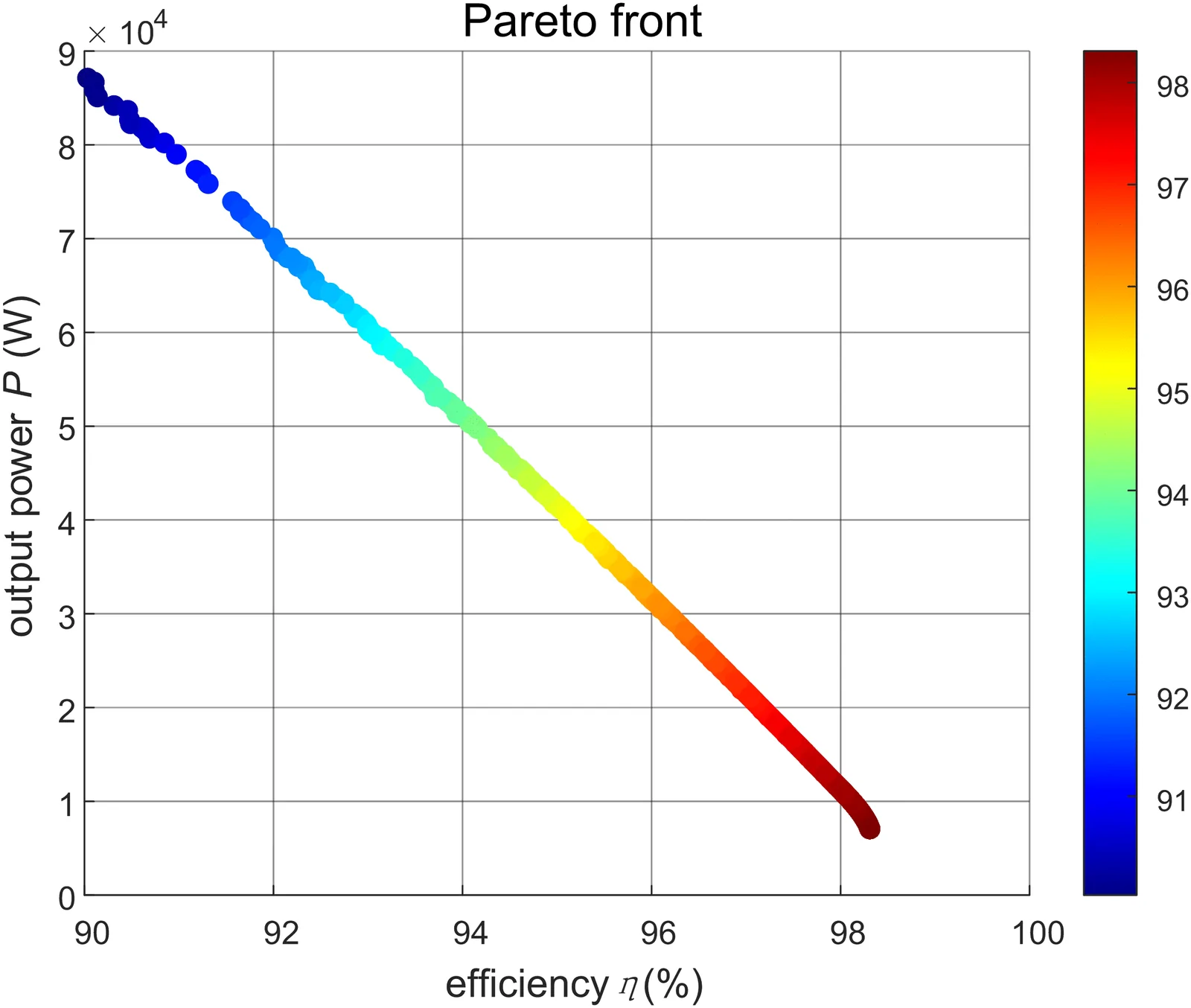
Pareto front of the LCC-S WPT system obtained using the VPSR-PSO multi-objective optimization algorithm.

Fig 12 shows the expected trade-off between output power and transmission efficiency. Toward the high-power end of the front, current and modeled losses increase and efficiency decreases. At the high-efficiency end, less power is transferred. The Pareto front therefore provides alternative parameter sets rather than a single optimum. Three points from different parts of this front are examined in the following subsection.

### 4.5. Simulation evaluation of representative Pareto-front solutions

To further examine the model-level dynamic behavior and load-side electrical stress of the Pareto-front solutions, three representative points were selected from the front for Simulink-based simulations. The corresponding parameters and system performance indicators are summarized in Table 10.

**Table 10:** Selected Pareto-front solutions.

Group	$L_{P}$ (μH)	$R_{L}$ (Ω)	M (μH)	*η* (%)	$P_{out}$ (W)
1	11.69	36.18	57.35	90.00	87,286
2	19.00	41.85	60.00	95.61	35,277
3	38.76	33.02	60.00	98.00	11,258

As shown in Table 10, the three solutions represent different locations along the obtained Pareto front. Group 1 is a high-power boundary candidate, providing 87.286 kW at an efficiency of 90.00%. Group 2 represents an intermediate power–efficiency trade-off, with 35.277 kW and 95.61% efficiency, whereas Group 3 represents the high-efficiency end of the investigated front, with 11.258 kW and 98.00% efficiency. It should be noted that Groups 2 and 3 both reach the prescribed upper mutual-inductance bound of *M* = 60μH. They should therefore be interpreted as boundary-constrained candidates within the present search domain rather than as evidence of an unconstrained interior optimum with respect to magnetic coupling.

It should be emphasized that the power levels in Table 10 are obtained under the specified model assumptions and parameter ranges and should be interpreted as Pareto-front design candidates rather than final hardware-rated operating points. The first solution represents a high-power boundary candidate and would require dedicated hardware design, including inverter and compensation-component ratings, coil structure, insulation design, thermal management, electromagnetic safety, and protection circuits. The second and third solutions represent intermediate and high-efficiency model-based candidates, respectively, and are retained for subsequent prototype-level evaluation rather than direct practical deployment.

To further clarify the practical meaning of the reported power levels, Table 11 provides additional model-calculated electrical-stress indicators. The RMS currents were calculated according to the equivalent-circuit model in Eqs. (1)–(5), while the estimated model loss was obtained from the difference between input and output power.

**Table 11:** Model-level electrical-stress indicators of the selected Pareto-front solutions.

Group	$P_{out}$ (W)	*η* (%)	$P_{in}$ (W)	$I_{1,rms}$ (A)	$I_{2,rms}$ (A)	Estimated total model loss (W)
1	87,286	90.00	96984	58.260	49.118	9698
2	35,277	95.61	36897	38.054	29.033	1620
3	11,258	98.00	11488	19.113	18.465	230

As shown in Table 11, the high-power boundary solution corresponds to higher RMS current and greater estimated model loss than the other two solutions. Therefore, this point should be interpreted as an upper-bound Pareto candidate under the specified model assumptions rather than a directly deployable hardware operating point. In contrast, the balanced and high-efficiency solutions exhibit lower modeled current stress and estimated losses, making them more appropriate candidates for subsequent prototype-level evaluation. Detailed current density, conductor selection, insulation design, and thermal management depend on the specific coil geometry and hardware implementation, and therefore are not treated as independent optimization targets in the present model-based study.

To provide an intuitive comparison of the dynamic characteristics corresponding to the three Pareto solutions, Fig 13 presents the input and output voltage and current waveforms of the LCC-S system under each parameter set. It should be noted that the input voltage shown in Fig 13 is the square-wave output voltage of the high-frequency inverter, which is consistent with the input voltage *u*_in_ defined in Fig 2. In the analytical model, the dominant fundamental-frequency response of this square-wave excitation is used for impedance-based calculation, whereas in the Simulink switching model the complete square-wave voltage is directly applied. Therefore, the square-wave voltage in Fig 13 is not inconsistent with Fig 2; instead, Fig 13 provides the time-domain switching evaluation of the inverter-excited WPT system. The input current corresponds to the current flowing into the primary compensation network, while the output voltage and output current correspond to the voltage across the secondary-side load $R_{L}$ and the current in the secondary-side circuit, respectively. Figs 13(a)–(b) show the input and output waveforms for the first high-power boundary candidate; Figs 13(c)–(d) correspond to the second solution balancing power and efficiency; Figs 13(e)–(f) depict the waveforms for the third high-efficiency candidate. It can be observed that, as the parameter combinations vary, the amplitudes of the system voltages and currents change significantly.

**Fig 13:**
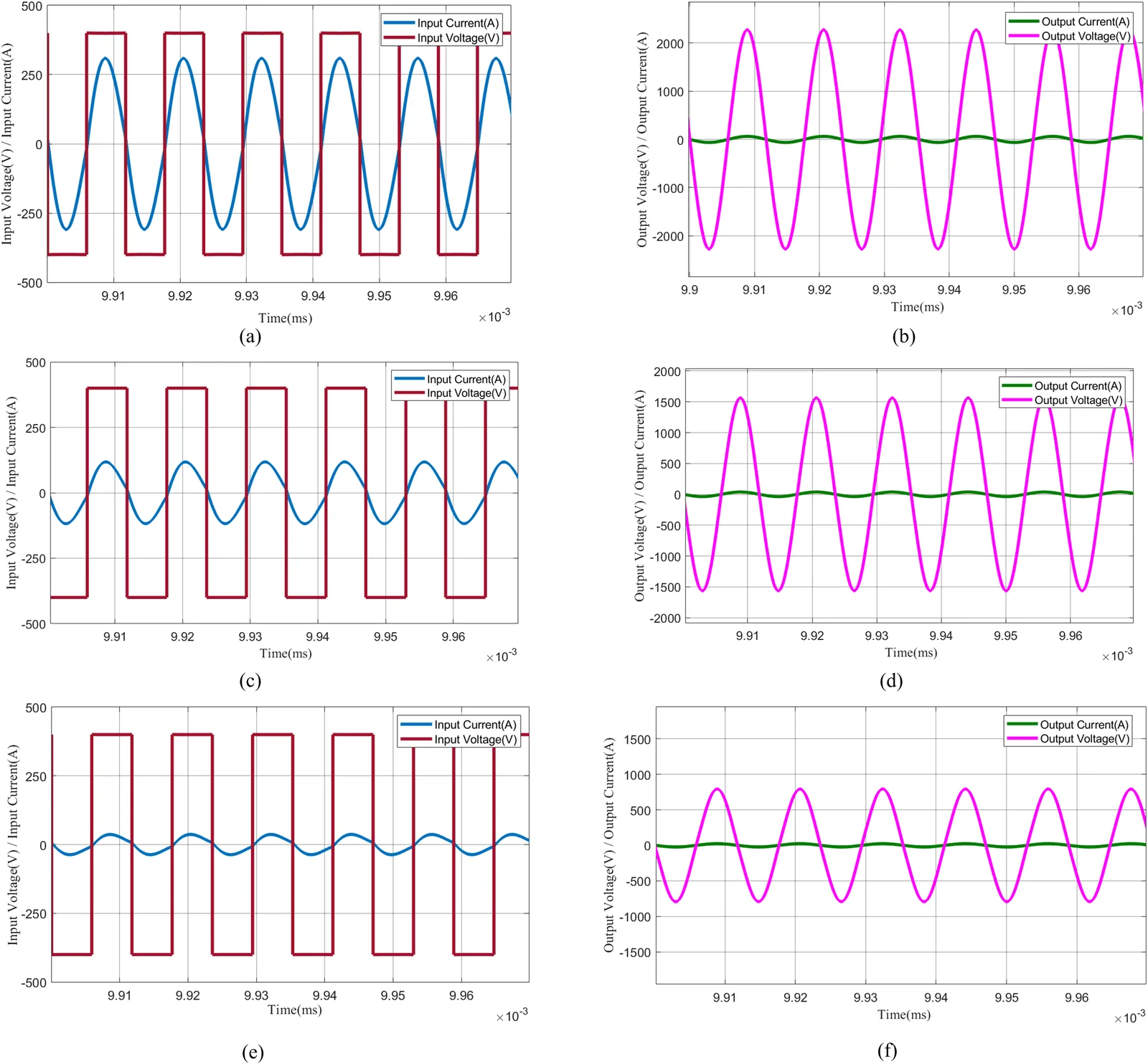
Time-domain switching simulation results of the LCC-S wireless power transfer system under three representative Pareto-front candidates: (a–b) high-power boundary candidate, (c–d) balanced power-efficiency candidate, and (e–f) high-efficiency candidate.

The switching-level Simulink results are lower than the corresponding analytical values for all three representative points. For Group 1, the analytical model gives $P_{\text{out}}=87.286\text{kW}$ and η=90\%, whereas the Simulink model gives $P_{\text{out}}=80.230\text{kW}$ and η=83\%, corresponding to an output-power difference of 8.08% and an efficiency difference of 7.00 percentage points. For Group 2, $P_{\text{out}}$ decreases from 35.277 to 32.212 kW, corresponding to an 8.69% difference, while efficiency decreases from 95.61% to 95.10%, a difference of 0.51 percentage points. For Group 3, $P_{\text{out}}$ decreases from 11.258 to 10.018 kW, corresponding to an 11.01% difference, while efficiency decreases from 98.00% to 97.70%, a difference of 0.30 percentage points. In the Simulink evaluation, output power is calculated from the RMS voltage and RMS current across the equivalent resistive load, consistent with the load-side power definition used in the analytical model.

These discrepancies arise from the different modeling levels used in the two evaluations. The optimization kernel employs a fundamental-harmonic approximation to enable repeated evaluation of a large number of candidate parameter sets, whereas the switching-level Simulink model retains the complete inverter square-wave excitation and the associated harmonic components. The relatively larger efficiency discrepancy for Group 1 coincides with its substantially higher current and modeled loss, as shown in Table 11, indicating greater sensitivity of the high-power boundary point to differences between the analytical and switching-level representations. The switching simulation therefore provides an additional model-level consistency check of the Pareto candidates rather than experimental validation.

Overall, the analytical and switching-level results show consistent power–efficiency trade-off trends across the three representative candidates, although quantitative discrepancies remain between the two modeling levels. The selected points therefore illustrate different locations along the model-derived Pareto front rather than experimentally verified operating points. These results support the use of the proposed framework for preliminary parameter screening within the specified model assumptions, while hardware-specific validation remains necessary before practical operating points are established.

### 4.6. Model limitations and practical feasibility discussion

All results reported in this study are model-based. The analytical optimization kernel employs the FHA and does not explicitly model several hardware-dependent effects, including inverter dead time and device voltage drops, semiconductor conduction and switching losses, tolerances and temperature dependence of compensation components and winding resistances, magnetic-core and frequency-dependent winding losses, or measurement uncertainty [25,27,29]. In addition, coil displacement or angular misalignment changes the mutual inductance and may also alter the coil self-inductances and loss characteristics. These effects can shift both output power and efficiency relative to the model-derived Pareto candidates and should be included when the framework is applied to a specific prototype.

FHA was retained because it makes repeated objective evaluation practical during optimization. The Simulink model keeps the complete square-wave excitation and provides a more detailed time-domain representation, but it is still a simulation model. As quantified in Section 4.5, the output-power differences between the analytical and switching-level models range from 8.08% to 11.01%, while the corresponding efficiency differences range from 0.30 to 7.00 percentage points. These discrepancies reflect the different levels of waveform representation used by the FHA-based analytical model and the complete switching model. A recent comparison of lumped-parameter and distributed electromagnetic WPT models also reported that computationally inexpensive lumped models can lose accuracy when distributed effects become important [40].

The occurrence of *M* = 60 μH in two representative solutions further indicates that part of the obtained Pareto front is influenced by the prescribed magnetic-coupling boundary. Extending the numerical upper bound alone would not necessarily constitute a physically meaningful sensitivity test because the attainable mutual inductance is determined by a specific coil geometry, air gap, alignment condition, and magnetic structure. Since these hardware quantities are not independently parameterized in the present equivalent-circuit optimization, the current results are deliberately interpreted only within the specified *M* = 1–60μH domain. Future prototype-oriented optimization should first determine an experimentally or electromagnetically validated coupling interval for the selected coupler and then repeat the Pareto analysis within that hardware-specific range.

The Pareto points should therefore be treated as preliminary model-based design candidates rather than directly deployable operating points. For a specific prototype, the ranges of *L*_p_, *R*_L_ and particularly *M* should first be restricted according to component ratings, coupler geometry, nominal coil spacing, allowable misalignment, and thermal and electromagnetic constraints. A candidate can then be selected according to the required power–efficiency trade-off and subsequently verified using a hardware prototype. Such validation should evaluate component tolerances, coil displacement, temperature-dependent losses, inverter non-idealities, semiconductor losses, and magnetic losses that are not fully represented in the present optimization model.

## 5. Conclusion

We formulated the joint selection of *L*_*p*_, *R*_*L*_, and *M* in an LCC-S WPT system as a two-objective parameter-design problem involving output power and transmission efficiency. The optimization framework integrates dynamic inertia adjustment, velocity pausing, and stagnation reset within an archive-based PSO rather than introducing fundamentally new general-purpose optimization operators. Across 10 independent benchmark runs, VPSR-PSO increased mean HV by 8.45%, reduced Spacing by 41.14%, and reduced the observed MATLAB runtime by 26.30% relative to the baseline MOPSO. Statistical testing confirmed the improvement over MOPSO, whereas NSGA-II and MOGWO achieved better final Pareto-quality indicators but showed longer observed runtimes under the reported implementation. The ablation analysis further showed that velocity pausing and stagnation reset provide the main performance improvements, while nonlinear dynamic inertia primarily contributes to search stability rather than a clear independent improvement in final Pareto-front quality.

The resulting Pareto front provides model-based candidates corresponding to high-output-power, intermediate power–efficiency, and high-efficiency regions. Switching-level Simulink evaluation showed consistent trade-off trends while also revealing quantitative discrepancies from the FHA-based analytical predictions. The proposed framework should therefore be interpreted as an application-specific tool for preliminary LCC-S parameter screening and power–efficiency trade-off analysis. The reported candidates are not experimentally validated operating points, and prototype-level verification remains necessary before hardware implementation.

### 5.1. Declaration of generative AI and AI-assisted technologies in the writing process

During the preparation of this manuscript, the authors used ChatGPT (OpenAI) to assist with English-language editing, including grammar correction, sentence restructuring, and improvement of readability. The scientific content was initially prepared by the authors in Chinese and subsequently translated and revised by the authors before AI-assisted language polishing. The AI tool was not used to generate research data, mathematical models, simulation results, or scientific conclusions. All AI-assisted revisions were reviewed and edited by the authors, who take full responsibility for the final content of the manuscript.

## Supporting information

S1 Code MATLAB source code used for VPSR-PSO optimization, benchmark algorithms, repeated-seed parameter analyses, ablation analysis, and performance and statistical metric evaluation. The archive includes the MATLAB scripts used to generate and evaluate the principal optimization results reported in the manuscript.
(ZIP)

S1 Data Numerical data underlying the results reported in the manuscript. The archive contains run-level benchmark data, repeated-seed parameter-analysis data, ablation results, convergence data, Pareto-front source data, and numerical values underlying the analytical and switching-level comparisons.
(ZIP)
